# Thermally activated delayed fluorescence in a deep red dinuclear iridium(iii) complex: a hidden mechanism for short luminescence lifetimes†

**DOI:** 10.1039/d3sc04450e

**Published:** 2023-11-14

**Authors:** Piotr Pander, Andrey V. Zaytsev, Amit Sil, Glib V. Baryshnikov, Farhan Siddique, J. A. Gareth Williams, Fernando B. Dias, Valery N. Kozhevnikov

**Affiliations:** a Faculty of Chemistry, Silesian University of Technology M. Strzody 9 44-100 Gliwice Poland piotr.pander@polsl.pl; b Centre for Organic and Nanohybrid Electronics, Silesian University of Technology Konarskiego 22B 44-100 Gliwice Poland; c Department of Physics, Durham University South Road Durham DH1 3LE UK f.m.b.dias@durham.ac.uk; d Department of Applied Sciences, Northumbria University Newcastle upon Tyne NE1 8ST UK valery.kozhevnikov@northumbria.ac.uk; e Department of Chemistry, Durham University South Road Durham DH1 3LE UK j.a.g.williams@durham.ac.uk; f Laboratory of Organic Electronics, Department of Science and Technology, Linköping University SE-60174 Norrköping Sweden; g Department of Pharmaceutical Chemistry, Faculty of Pharmacy, Bahauddin Zakariya University Multan 60800 Pakistan

## Abstract

The high luminescence efficiency of cyclometallated iridium(iii) complexes, including those widely used in OLEDs, is typically attributed solely to the formally spin-forbidden phosphorescence process being facilitated by spin–orbit coupling with the Ir(iii) centre. In this work, we provide unequivocal evidence that an additional mechanism can also participate, namely a thermally activated delayed fluorescence (TADF) pathway. TADF is well-established in other materials, including in purely organic compounds, but has never been observed in iridium complexes. Our findings may transform the design of iridium(iii) complexes by including an additional, faster fluorescent radiative decay pathway. We discover it here in a new dinuclear complex, 1, of the form [Ir(*N^C*)_2_]_2_(μ-L), where *N^C* represents a conventional *N^C*-cyclometallating ligand, and L is a bis-*N^O*-chelating bridging ligand derived from 4,6-bis(2-hydroxyphenyl)-pyrimidine. Complex 1 forms selectively as the *rac* diastereoisomer upon reaction of [Ir(*N^C*)_2_(μ-Cl)]_2_ with H_2_L under mild conditions, with none of the alternative *meso* isomer being separated. Its structure is confirmed by X-ray diffraction. Complex 1 displays deep-red luminescence in solution or in polystyrene film at room temperature (*λ*_em_ = 643 nm). Variable-temperature emission spectroscopy uncovers the TADF pathway, involving the thermally activated re-population of S_1_ from T_1_. At room temperature, TADF reduces the photoluminescence lifetime in film by a factor of around 2, to 1 μs. The TADF pathway is associated with a small S_1_–T_1_ energy gap Δ*E*_ST_ of approximately 50 meV. Calculations that take into account the splitting of the T_1_ sublevels through spin–orbit coupling perfectly reproduce the experimentally observed temperature-dependence of the lifetime over the range 20–300K. A solution-processed OLED comprising 1 doped into the emitting layer at 5 wt% displays red electroluminescence, *λ*_EL_ = 625 nm, with an EQE of 5.5% and maximum luminance of 6300 cd m^−2^.

## Abstract 2

Thermally activated delayed fluorescence (TADF) is found to play an important role in accelerating the rate of radiative decay in luminescent iridium(iii) complexes, shown in this instance for dinuclear complexes formed with high stereoselectivity.

## Graphical abstract

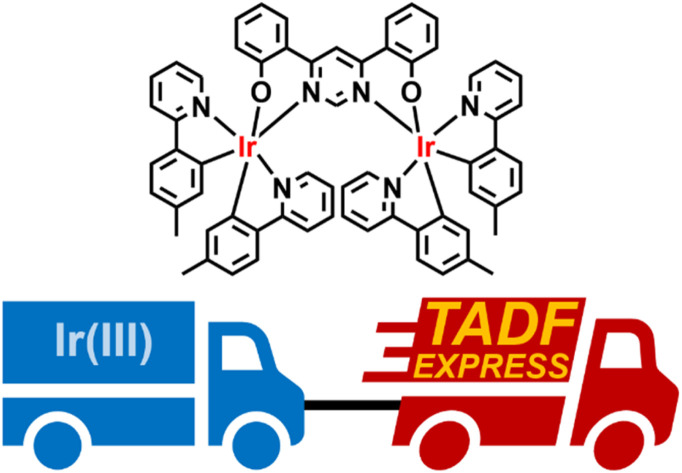

## 1 Introduction

Phosphorescent complexes of iridium(iii) and platinum(ii) have become widely used luminescent dopants in OLEDs in recent years, thanks to their high stability and reliability.^1–5^ The archetypal example is the green-emitting, tris-cyclometallated Ir(iii) complex *fac*-Ir(ppy)_3_ (ppyH = 2-phenylpyridine). The triplet radiative rate constants (*k*^T^_r_) of such metal complexes are generally several orders of magnitude larger than those of metal-free room-temperature phosphors.^6^ The *k*^T^_r_ value depends on the spin–orbit coupling (SOC) between the singlet and triplet states, which is in turn determined by the extent to which metal centred d orbitals contribute to the lowest excited states.^7^ In order to red-shift the emission to obtain red- and near-infrared (NIR)-emitting metal complexes, organic ligands featuring more extended conjugation are required, but this is typically accompanied by a reduction in metal admixtures, with predominantly ligand-centred character to the lowest excited states.^8,9^ The *k*^T^_r_ value suffers as a result,^8,10^ limiting the luminescence efficiency that is achievable. Recently, it has been discovered that certain dinuclear complexes of platinum(ii)^11,12^ and iridium(iii)^13^ show faster triplet radiative decay, thanks to more efficient mixing of singlet character into the emissive triplet state. The dinuclear structure of such platinum(ii) complexes is associated with a reduction in the energy gap between lowest singlet and triplet states (Δ*E*_ST_), leading to a pronounced thermally activated delayed fluorescence (TADF) contribution to the emission at room temperature.^14–16^ The further acceleration of radiative decay rates through TADF – regardless of the amplitude of spin–orbit coupling (SOC) induced by the metal centres^17,18^ – also has potentially major significance to the design of blue phosphors, as faster radiative decay helps to attenuate the pathways of emitter degradation in an OLED.

The relative values of phosphorescence *k*^T^_r_ and fluorescence *k*^S^_r_ decay rates, as well as the magnitude of Δ*E*_ST_, determine the extent of involvement of TADF at a given temperature.^1,14^ While increasing *k*^S^_r_ and reducing Δ*E*_ST_ generally promotes the thermally activated mechanism,^1,6,19^ delayed fluorescence will only be apparent if it leads to a faster decay pathway than *via* the normal phosphorescence (T_1_ → S_0_) route. For this reason, TADF is most commonly found in luminophores displaying long phosphorescence lifetimes, such as in metal-free molecules^20,21^ or metal complexes with relatively weak SOC. Contribution of the TADF mechanism to the luminescence is apparent in some complexes of Cu(i),^19,22,23^ Ag(i),^24^ Au(i),^25^ Au(iii),^26–28^ Pd(ii),^17,29,30^ Pt(ii),^14–16^ and also Zn(ii),^31^ W(vi)^32^ or Sn(iv)^33^ complexes where the triplet radiative decay lifetimes span from several microseconds to milliseconds. In Ir(iii) complexes, on the other hand, the level of S–T mixing is generally larger than in the former examples, leading to faster triplet decay rates, such that any TADF contribution may be easily overlooked. One study has reported some Ir(iii) complexes^34^ that demonstrate behaviour consistent with that observed in Pt(II) emitters featuring a TADF contribution,^16^ although TADF *per se* was not identified as the underlying mechanism.

**Scheme 1:**
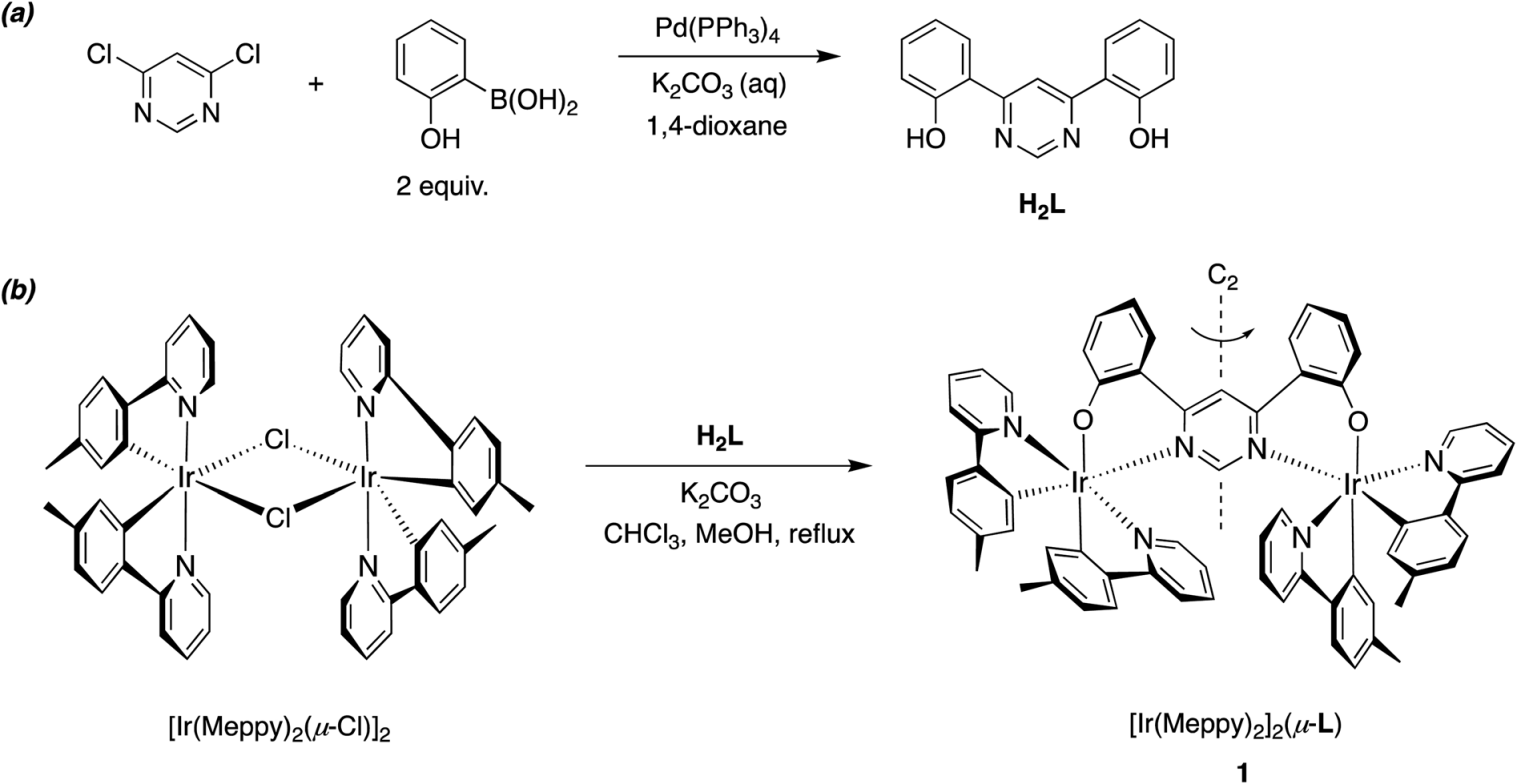
Synthesis of (a) the bridging proligand H_2_L from 4,6-dichloropyrimidine and 2-hydroxy-benzeneboronic acid (2 equiv.), and (b) the new dinuclear iridium complex *rac*-[Ir(Meppy)_2_]_2_(μ-L) (which will be referred to as 1; only one of the constituent enantiomers is shown) upon treatment of [Ir(Meppy)_2_(μ-Cl)]_2_ with H_2_L.

In this work, we demonstrate unequivocal evidence for the role of TADF in actively accelerating radiative decay in the new, red-emitting, dinuclear iridium(iii) complex 1 (Scheme 1). The TADF leads to a reduction in the room-temperature radiative decay lifetime of 1 from ∼2 to ∼1 μs. Our study demonstrates that TADF may indeed lead to the shortening of radiative decay lifetimes of Ir(iii) complexes, an effect which would otherwise be incorrectly attributed solely to phosphorescence. The application of such an Ir(iii) complex in an OLED is presented, where TADF is used for the first time to accelerate the radiative rate.

## 2 Molecular design, synthesis, and structure of 1

A range of dinuclear complexes incorporating two cyclometallated iridium(iii) centres have been reported.^35^ In some cases, for example, those where archetypal Ir(*N^C*)_3_ or [Ir(*N^C*)_2_(*N^N*)]^+^ units are connected through 1,4-substituted phenyl rings attached to the *N^C* or *N^N* ligands, the extent of communication between the metal centres is relatively minimal.^36–38^ The excited-state properties are then typically similar to those of the corresponding individual constituent metal complexes, albeit with energy-transfer potentially occurring between them when the ligand sets differ such that the excited-state energies associated with the two units are different. In contrast, the coordination of both metal ions simultaneously to a single, bridging aromatic heterocycle (*e.g.*, a pyrimidine or pyrazine) may lead to distinct properties, due to the strong perturbation of the orbital parentage of the pertinent low-energy excited states.^13,34,39–42^ Our previous work has employed bis-*N^C*-coordinating ligands based on 4,6-diarylpyrimidines and bis-aryl-substituted thiazolo[5,4-*d*]thiazoles, to bridge two cyclometallated Ir(iii) centres.^13,43^ In the present work, we explored an alternative bridge, namely the bis-*N^O*-coordinating ligand L derived from 4,6-bis(2-hydroxyphenyl)pyrimidine H_2_L, which bridges two Ir(*N^C*-Meppy)_2_ units in the new complex 1 {MeppyH = 2-(*p*-tolyl)pyridine} (Scheme 1).

The requisite ditopic, bridging proligand H_2_L was synthesized in 67% yield by the palladium-catalysed cross-coupling of 4,6-dichloropyrimidine with 2-hydroxybenzene boronic acid (Scheme 1). Subsequent treatment of the dichloro-bridged complex [Ir(Me_2_ppy)_2_(μ-Cl)]_2_ with H_2_L in the presence of base (K_2_CO_3_), in a mixture of chloroform and methanol at reflux, gave the desired dinuclear complex 1 in 68% yield after purification by column chromatography. These conditions are notably milder than those used to introduce bis-*N^C* bridges, where the activation energy associated with cyclometallation necessitates higher temperatures and the use of Ag^+^ ions to scavenge liberated chloride. The identity and the purity of the complex were confirmed by ^1^H and ^13^C NMR spectroscopy, elemental analysis and, subsequently, by X-ray crystallography (*vide infra*).

Due to the intrinsic *C*_2_ (or *D*_3_) chirality of bis- and tris-bidentate Ir(iii) complexes, dinuclear compounds based on Ir(*N^C*)_2_ {or Ir(*N^C*)_3_} units may comprise of a mixture of *meso* (ΔΛ) and *rac* (ΛΛ, ΔΔ) diastereomers. That can be problematic, as the diastereomers may display different properties from one another. The use of enantiomerically pure mononuclear building blocks can circumvent the problem, but such chiral separation is often difficult and not feasible on larger scales, and the starting configurations must also be retained under the conditions required to introduce the bridge. Another strategy that has been used to avoid the formation of diastereomeric mixtures is to employ non-stereogenic metal centres wherein the metal ions are coordinated by symmetric tridentate ligands.^34^

Nevertheless, in some binuclear systems comprising two bis-bidentate units – especially when a short linker is used such that steric interactions between the units influences the relative stabilities of the products – the formation of one diastereomer may occur diastereoselectively, or even diastereospecifically. Such is the case, for example, in the synthesis of the well-known chloro-bridged dimers of the form [Ir(*N^C*)_2_(μ-Cl)]_2_.^44^ The product is uniquely the *rac* pair (ΛΛ and ΔΔ). The *meso* (ΔΛ) product is apparently disfavoured through steric interactions of the *N^C* ligands on neighbouring metal centres, owing to their proximity.

**Fig. 1:**
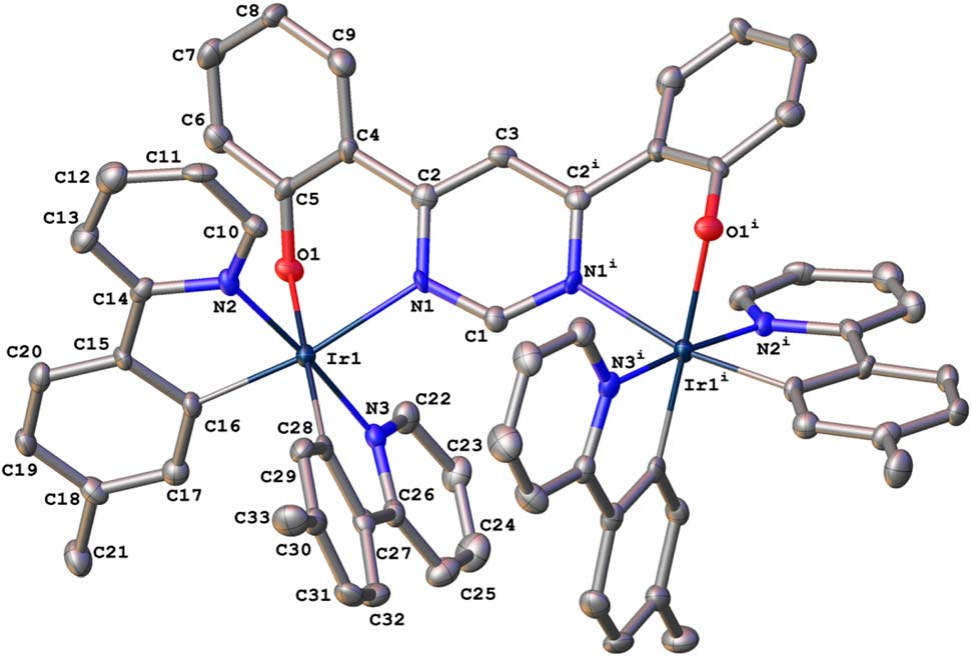
The molecular structure of dinuclear complex 1 in the crystal.

In the synthesis of 1, our tentative expectation was that the short distance between the metal centres, dictated by the compact pyrimidine bridging unit, would similarly lead selectively to the *rac* diastereoisomer. And, indeed, only one diastereomer was isolated from the chromatography column, based on ^1^H and ^13^C spectroscopy. X-ray diffraction analysis of a crystal of 1 confirms the hypothesis. The molecular structure (Fig. 1; CCDC no. 2288521) shows the expected structure, with the two Ir(*N^C*)_2_ centres both *N^O*-coordinated by the bridging ligand. The molecule in the crystal is located on a 2-fold rotation axis along C1⋯C3 (see also Scheme 1), such that the two metal centres have the same configuration; *i.e.*, ΛΛ (the enantiomer shown in Fig. 1) or ΔΔ. The Ir⋯Ir distance is 6.086(2) Å. Apparently, then, the choice of compact bridging ligand ensures that the reaction proceeds with a level of diastereoselectivity such that the *meso* isomer is not formed in significant amounts. Note that the three heterocyclic nitrogen atoms around each Ir(iii) centre are coordinated in a meridional arrangement, with the two pyridine rings occupying positions *trans* to each other. This is the same as the configuration observed in the chloro-bridged dimers [Ir(*N^C*)_2_(μ-Cl)]_2_. Thus, the *mer* → *fac* rearrangement that typically occurs during the formation of Ir(*N^C*)_3_ complexes thermally – which necessitates higher temperatures than those used here – is not observed. In those cases, the *mer* isomer is destabilised relative to the *fac* owing to two of the strongly σ-donating cyclometallating rings being positioned *trans* to one another. In 1, the cyclometallated rings are not *trans* to one another. The outcome is essentially the same as what is usually observed for [Ir(*N^C*)_2_(*N^N*)]^+^ complexes when prepared under similarly mild conditions from the chloro-bridged dimers, but with the *N^O* unit in place of the *N^N*.

## 3 Solution-state photophysics

### 3.1 Absorption and steady-state emission spectra

**Fig. 2:**
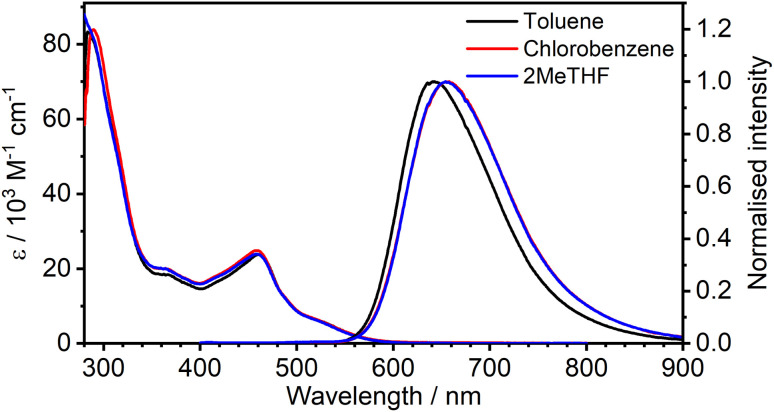
Absorption and photoluminescence (*λ*_ex_ = 365 nm) spectra of 1 in the three solvents indicated at a concentration of approximately 10^−5^ M.

The absorption and photoluminescence spectra of 1 are shown in Fig. 2, with corresponding numerical data compiled in Table 1. The absorption spectrum is quite typical of tris-cyclometallated iridium(iii) complexes in that it displays a set of quite intense bands of *ε* of the order of 10^4^ M^−1^ cm^−1^ in the visible region, attributed to charge-transfer transitions, as well as bands around 4× more intense in the far UV, associated with ligand-centred transitions. The visible-region bands are, however, substantially red-shifted compared to the related mononuclear complex Ir(Meppy)_3_, and the long-wavelength tail of 1 extends to around 600 nm compared to scarcely beyond 500 nm for Ir(Meppy)_3_. The red-shift is consistent with our previous work on pyrimidine-bridged dinuclear Ir(iii) and Pt(ii) complexes, attributed primarily to the lower-energy π* orbitals associated with the bis-coordinated pyrimidine that lead to correspondingly lower-energy ^1,3^MLCT transitions.

**Table 1:** Summary of the spectroscopic properties of 1 in the solvents indicated at 295 K

Solvent (dielectric constant *ε*)	Absorption *λ*_abs_^a^/nm (*ε*/M^−1^ cm^−1^)	Emission in deoxygenated solution at 295 K				
		*λ*_em_ b/nm	*Φ*_PL_ c	*τ* d/μs	*k*_r_ e/10^5^ s^−1^	*k*_nr_ e/10^5^ s^−1^
Toluene (2.4)	532sh (5200), 495sh (10 300), 462 (23 900), 369 (18 100), 284sh (83 700)	643	0.30	0.85	3.5	0.8
Chlorobenzene (5.6)	532sh (5200), 491sh (11 500), 460 (24 800), 369 (19 700), 290sh (84 100)	655	0.19	0.51	3.7	1.6
2MeTHF (7.0)	532sh (5000), 490sh (11 500), 458 (23 900), 363 (20 000), 289sh (82 000)	655	0.13	0.47	2.7	1.9

a Absorbance maxima and corresponding extinction coefficients.

b Emission maxima.

c Photoluminescence quantum yield recorded against rhodamine 6G in ethanol (*Φ*_PL_ = 0.91 (ref. 46)).

d Experimentally determined photoluminescence lifetime.

e Radiative *k*_r_ and non-radiative *k*_nr_ rate constants, estimated assuming that the emitting state is formed with unit efficiency such that *k*_r_ = *Φ*_PL_/*τ* and *k*_nr_ = (1 − *Φ*_PL_)/*τ*.

The complex displays deep red photoluminescence (PL), giving a broad, featureless spectrum in solution at room temperature, typical of Ir(iii)-based ^3^MLCT emitters. As in absorption, the emission is strongly red-shifted relative to the mononuclear analogue: *λ*_em_ = 655 nm for 1*versus* 510 nm for Ir(Meppy)_3_. There is no significant solvatochromism, neither in absorption nor emission, as is clear from Fig. 2 which shows the spectra in toluene and chlorobenzene superimposed on those in 2-MeTHF. The PL quantum yield of 0.30 in toluene is respectable for a deep-red emitter; values are slightly lower in the other two solvents (Table 1).

It is important to notice the clear overlap of the tail of the absorption band with the onset of the PL spectrum, around 550–580 nm. Such behaviour is not normally expected for a triplet emitter, if the lowest-energy absorption band of significant intensity is indeed a singlet, unless the S–T gap is very small (as would be required for a TADF contribution). Such overlap was the pointer to the TADF mechanism in the binuclear Pt(ii) complexes mentioned in the introduction.^14–16^ However, in the case of Ir(iii) complexes, the strength of singlet–triplet mixing through SOC is sufficient to enhance the oscillator strength of the normally strongly forbidden S_0_ → T_1_ transitions in the absorption spectrum, to the point that the bands have quite high molar absorptivities. The observation of pronounced overlap of absorption and emission is thus not, on its own, sufficient to infer a TADF contribution to the PL.

**Fig. 3:**
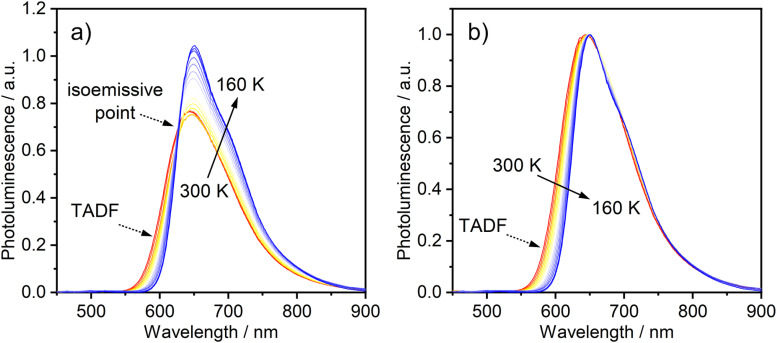
(a) PL spectra of 1 in toluene (*c* = 10^−5^ M) at various temperatures from 300 to 160 K. (b) The same set of spectra shown normalised at the *λ*_max_ values. *λ*_ex_ = 365 nm.

The temperature-dependence of the PL was recorded in toluene over the range 160–300 K. The evolution of the steady-state spectra with temperature is suggestive of two luminescent components: a higher-energy component, rather broad and featureless, which dominates at higher temperatures, and a lower-energy component with a discernible vibronic shoulder at lower temperatures (Fig. 3). A clear transition between the two spectral profiles is observed with a distinct iso-emissive point, indicating that the two components arise from two separate emissive states in equilibrium. This experimental picture is consistent with the behaviour of diplatinum(ii) complexes which display TADF,^14,16^ namely the lower-energy component being due to phosphorescence from T_1_ and the higher to fluorescence from S_1_, thermally populated from T_1_. Our attempts to deconvolve the luminescence spectra into separate phosphorescence and fluorescence spectra (which can be useful in understanding the thermodynamics^45^) were hampered by thermal broadening effects influencing the profiles at different temperature.

### 3.2 Time-resolved photoluminescence

**Fig. 4:**
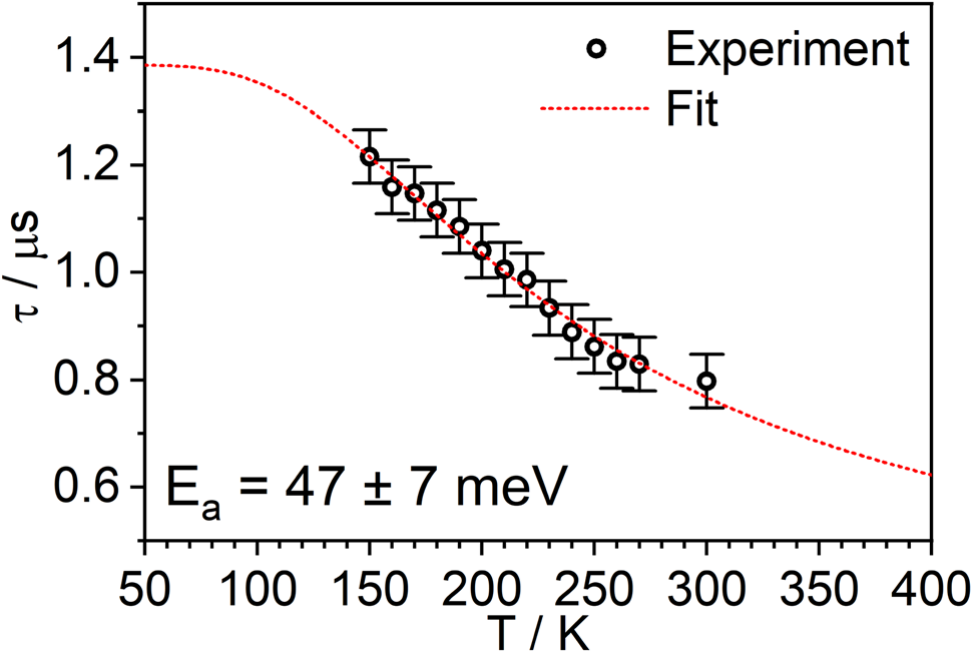
The experimentally observed PL decay lifetime of 1 (monitored at 640 nm) as a function of temperature (black circles) together with the best fit of eqn (1) to the data (red line).

The PL intensity of 1 displays mono-exponential decay, with lifetimes *τ*_obs_ in the range 0.47–0.85 μs at room temperature according to the solvent (Table 1). The variation of the experimentally observed lifetime with temperature *τ*_obs_(*T*) (Fig. 4) fits well to the model described by eqn (1),^1,47^ where Δ*E*_ST_ is the S_1_–T_1_ energy gap in J mol^−1^; *τ*_PH_ is the phosphorescence lifetime (s); *k*^S^_r_ is the radiative rate constant of S_1_ (s^−1^); *R* is the universal gas constant, 8.314 J mol^−1^ K^−1^; and *T* is the temperature in K. The best fit gives a value of Δ*E*_ST_ = 47 ± 7 meV and *k*^S^_r_ of (1.2 ± 0.2) × 10^7^ s^−1^ (corresponding to a natural radiative lifetime for S_1_ of about 83 ns). The Δ*E*_ST_ is a similar magnitude to that reported previously in a diplatinum(ii) TADF complex,^16^ while *k*^S^_r_ is consistent with values observed for charge-transfer singlet states in metal-free TADF emitters.^48^1
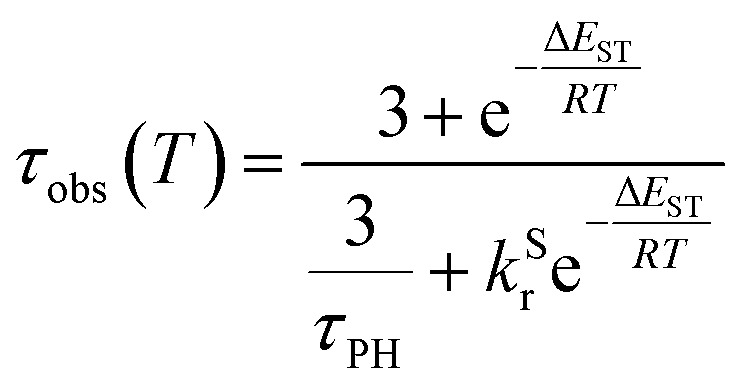


## 4 Solid-state photophysics

**Fig. 5:**
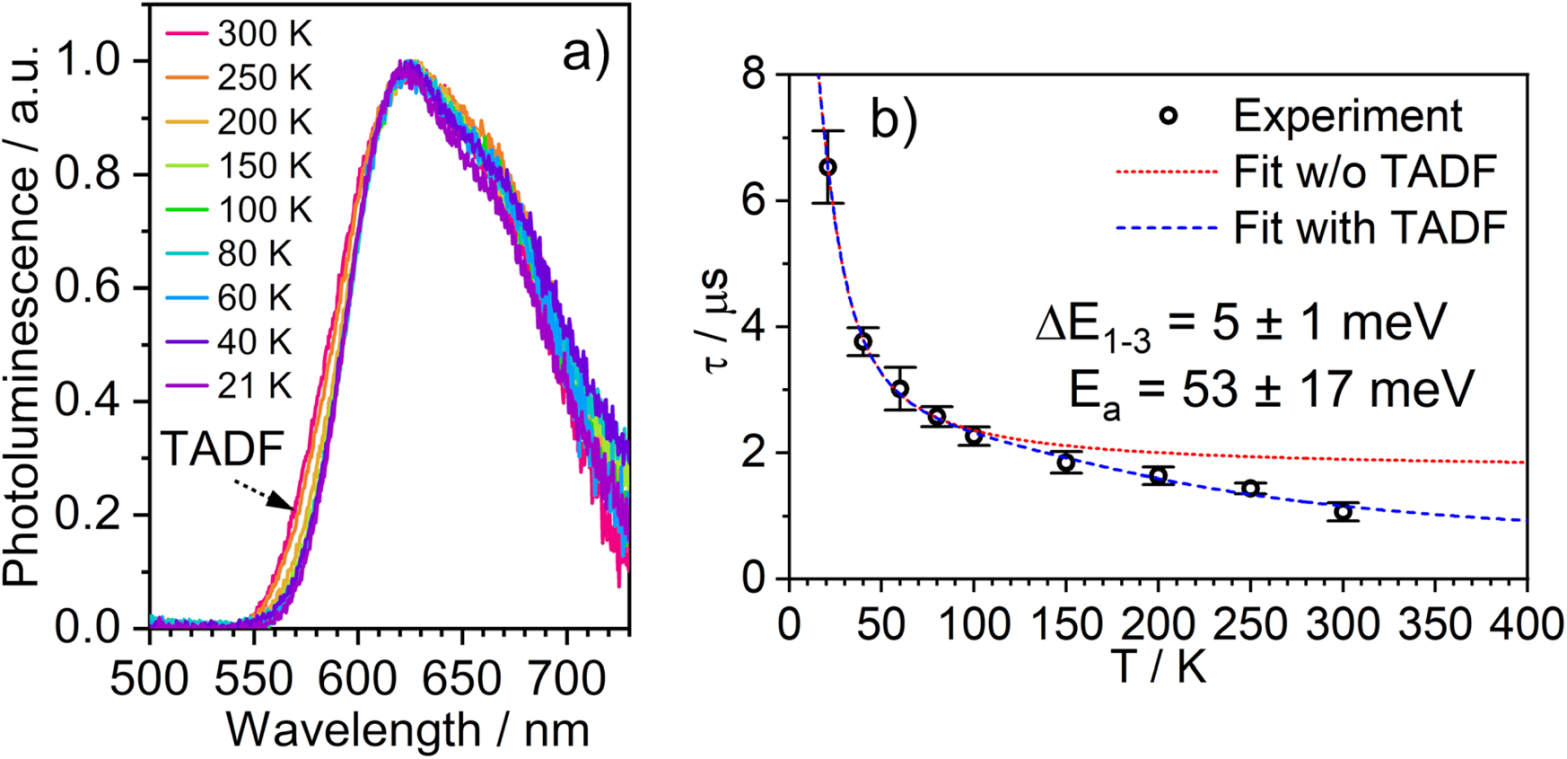
(a) The PL spectra of 1 in polystyrene matrix at (0.1% w/w) at the temperatures indicated. (b) The corresponding variation in *τ*_obs_. The experimental data points are represented by black circles and the best fit to eqn (2) by the dashed blue line. The corresponding “fit” in the absence of the TADF component is shown by the dotted red line. *λ*_ex_ = 355 nm.

The temperature dependence of the PL spectra and lifetimes of 1 in polystyrene film were investigated over the range 300–20 K (Fig. 5 and S5.4–5.6†). Access to the lower temperature range <80 K is insightful as it allows the splitting of the sublevels of T_1_ to be studied.^1,49^ The energy gap Δ*E*_1,3_ between sublevels 1 and 3 (Δ*E*_1,3_) – also known as the zero-field splitting (ZFS) – is often of the order of tens of cm^−1^ in 3rd row complexes,^1^ such that the thermal population of sublevel 3 influences the observed lifetime over the temperature range 20–80 K (Δ*E*_1,2_ is usually much smaller and so even lower temperatures <20 K are required to probe it). The magnitude of ZFS is associated with the strength of the SOC between the singlet and triplet manifolds, and a correlation has been observed between it and the phosphorescence radiative rate constant of metal complexes through the work of Yersin and co-workers in particular.^1^

The lifetime data in Fig. 5 are fitted to eqn (2), which takes into account the thermal equilibrium between the 1 and 3 sublevels of the lowest triplet state while assuming that Δ*E*_1,2_ is sufficiently small for the 1 and 2 sublevels to be considered equally populated over the temperature range used.2
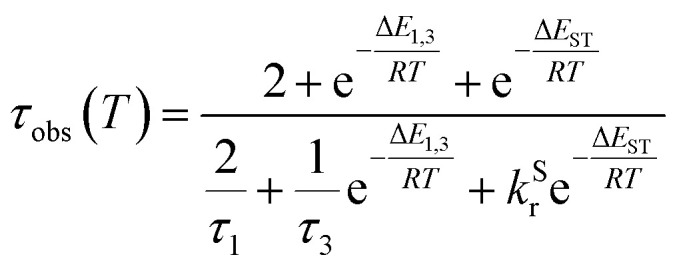


The PL lifetime of 1 increases only modestly from 1.1 μs at 300 K to 2.3 μs at 100 K, but then increases steeply to 6.5 μs at 21 K. Of course, it is the experimentally observed lifetime *τ*_obs_(*T*) that is measured, rather than the natural radiative lifetime. However, since the steady-state PL intensity remains essentially invariant over a wide range of temperatures including <100 K (Fig. S5.6†), it may be concluded that the variation in *τ*_obs_(*T*) is largely reflecting changes in the radiative rate as opposed to simple suppression of non-radiative decay. The initial increase in *τ*_obs_ can then probably be associated with the lowering influence of the TADF mechanism of re-population of S_1_ as the temperature is reduced, and then the subsequent larger increase is attributed to sublevel 3 being less thermally populated from sublevels 1 and 2 at the lowest temperatures. The experimental data give an excellent fit to eqn (2) (blue dashed line in Fig. 5b). On the other hand, if the data are fitted to a form of eqn (2) in which no TADF mechanism is included (*i.e.*, where Δ*E*_ST_ = ∞ and/or *k*^S^_r_ = 0), the fit is poor (red dotted line). The modelled difference between the two scenarios (*i.e.*, with or without TADF) suggests that the effect of delayed fluorescence is to halve the radiative decay lifetime of 1 at room temperature. The fit that incorporates TADF gives values of Δ*E*_ST_ = 53 ± 17 meV, Δ*E*_1,3_ = 5 ± 1 meV (=40 cm^−1^), and *k*^S^_r_ = (8 ± 5) × 10^6^ s^−1^ (or *τ*_S_ approx. 100 ns), values which are consistent with those obtained in toluene (*vide supra*). On the other hand, if the TADF mechanism is excluded, the ZFS would need to be 430 cm^−1^ to account for the behaviour. As that value is more than double the largest-ever recorded ZFS in Ir(iii) complexes with the highest contributions of MLCT character to the emitting states, it is improbable that efficient SOC alone could be responsible for the fast decay of 1.

Finally, we note that the time-resolved photoluminescence spectra reveal an invariance of the PL spectrum with time delay at any temperature (Fig. S5.5†), which confirms that the delayed fluorescence and phosphorescence have the same lifetime and thus that these two emissive states are in equilibrium. It also rules out intermolecular processes being responsible for delayed fluorescence. Collectively, the observations provide very strong evidence for TADF as the sole up-conversion mechanism.^47,50^

## 5 Calculations

Density functional theory (DFT) and time-dependent DFT (TD-DFT) as well as the quasi-degenerate perturbation theory (QDPT)^51,52^ with zeroth-order regular approximation (ZORA)^53,54^ implemented in Orca^55,56^ were used to gain additional insight into the luminescence mechanisms operating in 1. The ground state (S_0_) and triplet excited state (T_1_) geometries were optimised at the BP86 (ref. 57)/def2-TZVP^58^/CPCM(toluene) level of theory. Singlet and triplet radiative rate constants were calculated using the ZORA-corrected def2-TZVP basis sets^58^ for light atoms and a segmented all-electron, relativistically-contracted (SARC) def2-TZVP basis set for Ir.

**Fig. 6:**
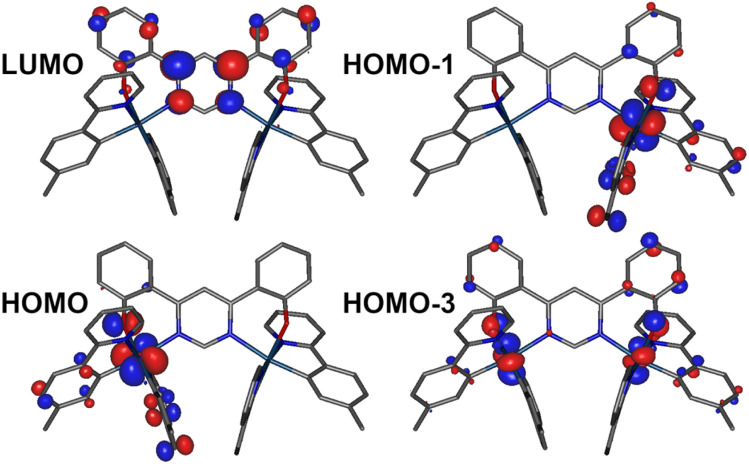
Relevant molecular orbital iso surfaces (iso = 0.05) for 1 calculated at the T_1_ geometry.

Spin–orbit coupled excited states (SOC states) are represented as mixed states with singlet and triplet admixtures. They are summarised in Table S4.1 and Fig. S4.2 in the ESI.† The lowest four SOC states are considered, which represent the triple-degenerate sublevels of the T_1_ (states 1 to 3), and the state 4, interpreted as S_1_. States 1 to 3 are dominated by T_1_ character with ∼10% admixtures from other states (mainly upper triplet states T_*n*_). State 4 is 81% S_1_ with triplet state admixtures. The orbital topology of the S_1_ and T_1_ states is therefore considered. S_1_ has dominant HOMO → LUMO character (>98%), while T_1_ is also dominated by the HOMO→LUMO transition (92%), but with small contributions from HOMO-1 → LUMO (4%) and HOMO-3 → LUMO (1%) (Fig. 6). The HOMO and HOMO-1 involve d orbitals on a different metal centre and ligand orbitals coordinating the respective metal ion: the oxygen p orbital of the phenolate ligand, as well as the phenyl π orbitals of the C,N ligands (Fig. 6). HOMO-3 is similar to HOMO and HOMO-1 but it involves both metal centres. The LUMO is localised on the diphenylpyrimidine bridge, as in Pt(ii) complexes containing related structural motifs.^14,16^ Such orbital topology of S_1_ and T_1_ states gives them a clear MLCT + IL (interligand) character. Since the two lowest excited states differ in orbital topology, a relatively large < T_1_|*H*_SO_|S_1_> = 91 cm^−1^ arises (*cf.* values of the order of ∼1 cm^−1^ expected in metal-free TADF emitters^59^), suggesting that direct S_1_ ↔ T_1_ ISC and reverse-ISC (RISC) should be fast. This does not exclude other states being involved in the RISC/ISC process, but rather indicates that the S_1_ ↔ T_1_ spin-flip may be sufficiently fast on its own to explain the experimental behaviour of the complex.

A relatively small splitting between the lowest SOC states is observed, with Δ*E*_1,2_ = 1 meV (or 8 cm^−1^) and Δ*E*_1,3_ = 4 meV (32 cm^−1^), indicating a rather weak splitting compared to, say, Ir(ppy)_3_ (for which Δ*E*_1,3_ = 85–170 cm^−1^).^1^ On the other hand, the value of Δ*E*_ST_ = Δ*E*_1,4_ = 69 meV (557 cm^−1^) is small and allows for thermal up-conversion from states 1–3 into the S_1_. The calculated singlet radiative rate constant *k*^S^_r_ is 4.9 × 10^6^ s^−1^, corresponding to a natural decay lifetime *τ*_0_^S^ of 205 ns. The summative triplet radiative rate constant at 295 K, *k*^T^_r_ is 3.1 × 10^5^ s^−1^, corresponding to a natural phosphorescence lifetime *τ*^T^_0_ of 3.2 μs. When the TADF contribution is included at *T* = 295 K, *k*_r_ increases to 4.2 × 10^5^ s^−1^ (the corresponding lifetime reduces to 2.4 μs). Thus, all of the calculated data describing the luminescence of 1 are in very good agreement with the experimental results.

**Fig. 7:**
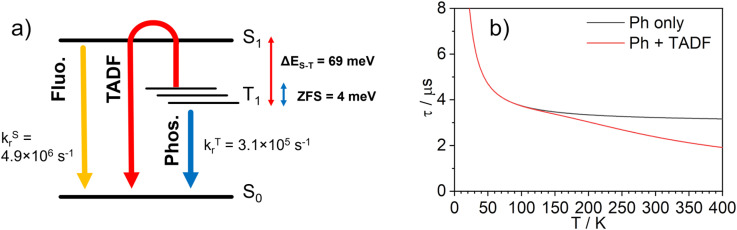
(a) Computational model of the photophysics of 1; (b) simulated natural lifetime, *τ*_0_, of 1 as a function of temperature obtained using eqn (3). The grey line represents the lifetime simulated without inclusion of a TADF component (*i.e.*, phosphorescence only, *k*^S^_r_ = 0 or Δ*E*_ST_ = ∞), and the red line the corresponding simulation in which the TADF component to the decay is included.

The temperature dependence of the luminescence lifetime of 1 has been modelled using eqn (3), which takes into account the radiative decay of all four of the SOC states, using the calculated energy gaps between them (here, 
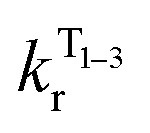
 represent the radiative rates of states 1 to 3). The plot of the calculated data (Fig. 7b) perfectly reproduces the observed changes in the photoluminescence lifetime, reflecting first the thermally activated occupation of the higher triplet sublevels at the lowest temperatures and then the population of the S_1_ (*i.e.*, the TADF contribution). The excellent agreement between the calculated and experimental data thus demonstrates the importance of the TADF mechanism to the fast luminescence decay of 1. Its behaviour at room temperature (indeed, any temperature above about 150 K) cannot be adequately explained without inclusion of the TADF contribution.3
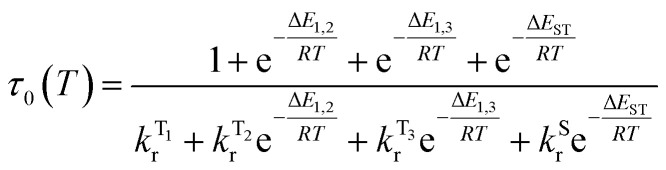


## 6 OLED devices

**Fig. 8:**
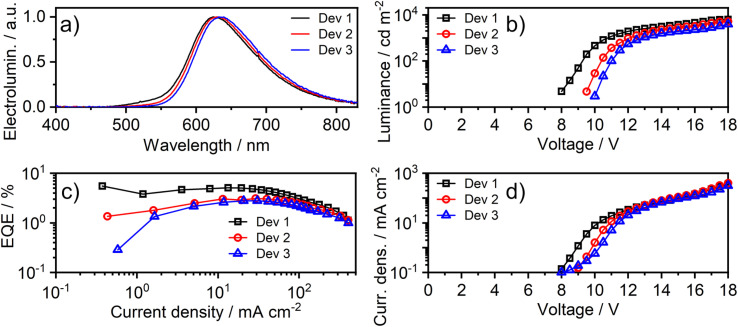
Characteristics of devices 1–3: (*a*) electroluminescence spectra; (b) luminance–voltage; (c) external quantum efficiency (EQE) *vs.* current density; (d) current density–voltage characteristics.

Fast radiative decay in the red region of the spectrum evidently renders complexes like 1 of great interest as emitters for deep-red OLEDs. Proof-of-concept, solution-processed OLEDs have therefore been fabricated in this work, using 1 as the luminescent dopant. The electrical and electroluminescent characteristics of the OLEDs are presented in Fig. 8, with pertinent numerical data in Table 2. The OLED structure used in this work has been adapted from our previous studies,^14,60^ but we used a different hole-transport material to better match the HOMO of the luminescent dopant: ITO|AI4083 (30 nm)|TCTA : PO-T2T (80 : 20) co. 1 (*x*%) (65 nm)|PO-T2T (50 nm)|LiF (0.8 nm)|Al (100 nm). It comprises of PEDOT AI4083 as the hole-injection layer; an emissive layer consisting of a blend host for 1 of TCTA {4,4′,4-tris(carbazol-9-yl)triphenylamine} as a hole-transport component and PO-T2T {2,4,6-tris[3-(diphenylphosphinyl)phenyl]-1,3,5-triazine} as an electron-transport component; PO-T2T as the electron-transport layer; LiF as the electron-injection layer; an Al cathode.

**Table 2:** Characteristics of OLED devices fabricated with 1 as the emitter. Device structure: ITO|AI4083 (30 nm)|TCTA : PO-T2T (80 : 20) co. 1 (*x*%) (65 nm)|PO-T2T (50 nm)|LiF (0.8 nm)|Al (100 nm)

Device	*x* a, %	*λ*_EL_, nm b	CIE 1931 (*x*,*y*)	*Φ*_PL_ c	EQE_max_^d^, %	Max. luminance, cd m^−2^	Max. radiant emittance, mW cm^−2^
Dev 1	5	625	(0.61, 0.38)	0.17 ± 0.03	5.5	6300	9.5
Dev 2	8	630	(0.63, 0.37)	0.16 ± 0.03	3.1	5200	8.7
Dev 3	12	635	(0.64, 0.36)	0.15 ± 0.03	2.8	4200	7.6

a Doping concentration of 1 by weight in the emissive layer.

b EL maxima.

c PL quantum yield of the emissive layer in a nitrogen atmosphere.

d Device maximum external quantum efficiency.

The effect of doping concentration on OLED properties has been studied, showing that low loads lead to higher efficiency. Interestingly, a distinct red-shift of the intensity-normalised electroluminescence (EL) spectrum is observed as the concentration of 1 increases; *e.g. λ*_EL_ = 625 and 635 nm at 5 and 12% w/w respectively. Given the overlap between the absorption and PL spectra observed in solution, it is likely that the self-absorption of EL within the emissive layer is the most likely reason for the apparent red-shift. The most efficient OLED Device 1 displays a maximum external quantum efficiency (EQE) of 5.5% and a maximum luminance of 6300 cd m^−2^.

## 7 Conclusions

We present here the first example of an iridium(iii) complex where a TADF mechanism has been demonstrated – both computationally and experimentally – to accelerate the luminescence decay significantly, and the first report of the use of such an emitter in an OLED. This work proves the concept that TADF can lead to an important, additional route to accelerating the luminescence decay of iridium(iii) complexes. It complements the spin–orbit coupling model based solely on partially allowed phosphorescence, which has hitherto always been assumed to be the only pathway for radiative decay of Ir(iii) materials. The mechanism leads to a *k*_r_ of 3.5 × 10^5^ s^−1^ at room temperature, a remarkably high value for such a deep red emitter new, and it arises from the small ∼50 meV S_1_–T_1_ gap. An OLED incorporating 1 displays an EQE of 5.5% with *λ*_EL_ = 625 nm.

In this work we present a vital, new luminescent mechanism in iridium(iii) complexes that may profoundly change the way one would design metal complexes with a short photoluminescence decay. Evidently, TADF can significantly shorten PL lifetimes even if not dominating the PL spectrum, and this feature can readily be exploited in multiple areas of research. Shortening the luminescence lifetimes of iridium(iii) complexes using our new strategy may be relevant, for example, to the development of blue and NIR OLEDs, where short decay times are crucial to reduced device degradation and increased efficiency of long-wavelength EL, respectively.

Our work extends the understanding of the possible emissive pathways available for iridium(iii) complexes and beyond the spin–orbit coupling-based model. TADF is likely to prove to be an overlooked pathway in metal complexes. It does not undermine the importance of the heavy atom effect and phosphorescence, but rather serves as a potentially powerful alternative strategy for designing more efficient luminescent complexes.

## Data availability

Our supporting research data is available from the Durham Research Online DATAsets Archive (DRO-DATA) open data repository. DOI: https://doi.org/10.15128/r2sb397833w.

## Author contributions

AVZ and PP contributed equally to this work. P.P. – conceptualization, formal analysis, investigation (photophysics, OLED devices, calculations, electrochemistry), visualization, writing – original draft, writing – review & editing; A. V. Z. – investigation (synthesis, characterisation); A. S. – investigation (crystallisation); G. V. B. – investigation (calculations); F. S. – investigation (calculations); J. A. G. W. – conceptualization, funding acquisition, project administration, supervision, writing – review & editing; F. B. D. – conceptualization, funding acquisition, project administration, resources, validation, writing – original draft, writing – review & editing; V. N. K. – conceptualization, funding acquisition, project administration, writing – original draft, writing – review & editing.

## Conflicts of interest

There are no conflicts of interest to declare.

## Supplementary Material

SC-014-D3SC04450E-s001

SC-014-D3SC04450E-s002
