# Supplementary material for: Thermally activated delayed fluorescence in a deep red dinuclear iridium(iii) complex: a hidden mechanism for short luminescence lifetimes
Source: Chem Sci. 2023 Nov 14;14(47):13934–43. doi: 10.1039/d3sc04450e (PMC10699548; doi:10.1039/d3sc04450e)
Supplement: SC-014-D3SC04450E-s001 [file SC-014-D3SC04450E-s001.pdf]

Electronic Supplementary Material (ESI) for Chemical Science.
This journal is © The Royal Society of Chemistry 2023

Supporting information

Thermally Activated Delayed Fluorescence in a Deep Red Dinuclear Iridium(III) Complex: a Hidden Mechanism for Short Luminescence Lifetimes

Piotr Pander,[a,b,c]* Andrey V. Zaytsev,[d] Amit Sil,[e] Glib V. Baryshnikov,[f] Farhan Siddique,[f,g] J. A. Gareth Williams,[e]* Fernando B. Dias[c]* and Valery N. Kozhevnikov[d]*

[a] *Faculty of Chemistry, Silesian University of Technology, M. Strzody 9, 44-100 Gliwice, Poland.*

[b] *Centre for Organic and Nanohybrid Electronics, Silesian University of Technology, Konarskiego 22B, 44-100 Gliwice, Poland.*

[c] *Department of Physics, Durham University, South Road, Durham, DH1 3LE, U.K.*

[d] *Department of Applied Sciences, Northumbria University, Newcastle upon Tyne, NE1 8ST, U.K.*

[e] *Department of Chemistry, Durham University, South Road, Durham, DH1 3LE, U.K.*

[f] *Laboratory of Organic Electronics, Department of Science and Technology, Linköping University, SE-60174 Norrköping, Sweden.*

[g] *Department of Pharmaceutical Chemistry, Faculty of Pharmacy, Bahauddin Zakariya University, Multan 60800, Pakistan.*

Table of contents

1. General....................2
2. Synthesis....................5
3. X-ray crystallography....................13
4. Theory....................17
5. Photophysics....................19
   a) Solution state....................19
   b) Solid film (polymer matrix)....................21
6. Electrochemistry....................24
7. OLED devices....................25
8. References....................26

1. General

Theory

We use density functional theory (DFT) and time-dependent DFT (TD-DFT) as well as the quasi-degenerate perturbation theory (QDPT)[1,2] with zeroth-order regular approximation (ZORA)[3,4] implemented in Orca 5.0.3[5,6] in order to gain an additional insight into the luminescence mechanisms in **1**. Ground state (S_0) and triplet excited state (T_1) geometries were optimised at the BP86[7]/def2-TZVP[8]/CPCM(toluene) level of theory which was found to be the most optimal for this task. Singlet and triplet radiative rates were calculated using ZORA-corrected def2-TZVP basis sets[8] for light atoms and a segmented all-electron relativistically contracted (SARC) def2-TZVP basis set for Ir. All molecular orbital (MO) iso surfaces were visualised using Gabedit 2.5.0.[9]

Geometry optimisations were performed at the BP86[7]/def2-TZVP[8] level of theory with def2/J[10] auxiliary basis set. Atom-pairwise dispersion correction with the Becke-Johnson damping scheme (D3BJ)[11,12] was included in the calculation. All geometries were verified to be true energy minima by a frequency calculation. All optimisations were performed with tight SCF and geometry convergence criteria. Excited state energy of TDDFT states was calculated using the resultant S_0 or T_1 geometry. In this case relativistically corrected triple-zeta basis sets with the zeroth-order regular approximation (ZORA)[3,4] were used: ZORA-def2-TZVP[8] with the SARC/J[13] auxiliary basis for all atoms except Ir for which a segmented all-electron relativistically contracted (SARC) SARC-ZORA-TZVP[13] basis set was used. Spin-orbit coupling (SOC) calculations were performed as implemented in the ORCA software. SOC matrix elements (SOCME) and SOC-corrected excitations (SOC states) were computed using the same settings as for the TDDFT states. The RI-SOMF(1X) setting was used to accelerate SOC calculations.

Electrochemistry

Cyclic voltammetry was conducted in a three-electrode, one-compartment cell. All measurements were performed using 0.1 M Bu_4NBF_4 (99%, Sigma Aldrich, dried) solution in dichloromethane (ExtraDry AcroSeal®, Acros Organics) as the supporting electrolyte. All solutions were bubbled with nitrogen prior to the measurement and the measurement itself was conducted in nitrogen atmosphere. Electrodes: working (Pt disc d = 1 mm), counter (Pt wire) and reference (Ag/AgCl calibrated against ferrocene) were used in the study. All cyclic voltammetry measurements were performed at room temperature with a scan rate of 50 mV s^{-1}.

Ionization potential (IP) and electron affinity (EA) are obtained from onset redox potentials; these figures correspond to HOMO and LUMO values, respectively. The ionization potential is calculated from onset oxidation potential $IP = E_{ox}^{CV} + 5.1$ and the electron affinity is calculated from onset

reduction potential EA = E_{red}^{CV} + 5.1.[14,15,16,17] An uncertainty of ±0.02 V is assumed for the electrochemical onset potentials.

Photophysics

Absorption spectra of 10^{-5} M solutions were recorded with UV-3600 double beam spectrophotometer (Shimadzu). Photoluminescence (PL) spectra of solutions and films were recorded using a QePro compact spectrometer (Ocean Optics) or FluoroLog fluorescence spectrometer (Jobin Yvon). Photoluminescence decays in film were recorded using nanosecond gated luminescence and lifetime measurements (from 400 ps to 1 s) using the third harmonic of a high-energy pulsed Nd:YAG laser emitting at 355 nm (EKSPLA). The emitted light was focused onto a spectrograph and detected with a sensitive gated iCCD camera (Stanford Computer Optics) having sub-nanosecond resolution. Time-resolved measurements were performed by exponentially increasing gate and integration times. Further details are available in reference.[18] Time-resolved decays in solution were recorded with a Horiba DeltaFlex TCSPC system using a 330 nm SpectraLED light source. Temperature-dependent experiments were conducted using a liquid nitrogen cryostat VNF-100 (sample in flowing vapour, Janis Research) under nitrogen atmosphere, while measurements at room temperature were recorded under vacuum in the same cryostat. Solutions were degassed using five freeze-pump-thaw cycles. Thin films in polystyrene were deposited from chloroform solutions. The films were fabricated through spin-coating and dried under vacuum at room temperature. Solid state emission spectra and photoluminescence quantum yields were obtained using an integrating sphere (Labsphere) coupled with a 365 nm LED light source and QePro (Ocean Optics) detector.

Determination of photoluminescence quantum yields in solution

Photoluminescence quantum yields were obtained using a gradient method in which we study relation (gradient) between the total photoluminescence intensity and absorbance at the excitation wavelength (same for both standard and analyte) in a range of concentrations for both analyte and standard – see equation below. We only consider data points with a constant gradient, so that the relation between photoluminescence intensity and absorbance is linear – indication of the photoluminescence yield being independent of concentration in this region. The eligible concentration range was 10^{-6}-10^{-5} M while absorbance of the standard was kept at below 0.05.

$$\Phi_x = \Phi_{standard}\left(\frac{grad_x}{grad_{standard}}\right)\left(\frac{\eta_x^2}{\eta_{standard}^2}\right)$$

Where: Φ_x, $\Phi_{standard}$ are the photoluminescence quantum yield of the analyte and standard, respectively; $grad_x$, $grad_{standard}$ are the gradients of the best linear fits of photoluminescence

intensity versus absorbance at λ_{ex} for the analyte and standard, respectively; η_x and $\eta_{standard}$ are the refractive indices of the solvents used for the analyte and standard, respectively.

OLED devices

OLEDs were fabricated by spin-coating / evaporation hybrid method. The hole injection layer (PEDOT AL4083), hole transport layer PVKH, and emitting layer (TCTA:PO-T2T + dopant) were spin-coated, whereas the electron transport layer (PO-T2T) and cathode (LiF/Al) were evaporated. We produced devices of 4 × 2mm pixel size. 2,4,6-Tris[3-(diphenylphosphinyl)phenyl]-1,3,5-triazine (PO-T2T, LUMTEC), tris(4-carbazoyl-9-ylphenyl)amine (TCTA, LUMTEC), poly(*N*-vinylcarbazole) (PVKH, Sigma Aldrich, M = 10^6 Da), LiF (99.995%, Sigma Aldrich), and aluminium pellets (99.9995%, Lesker) were purchased from the companies indicated in the parentheses. We used pre-patterned indium-tin-oxide (ITO) on glass substrate with a sheet resistance of 20 Ω cm^{-2} and ITO thickness of 100 nm. The substrates were cleaned by sonicating in acetone and subsequently in isopropanol for 15 minutes each and then treated with oxygen plasma for 6 minutes at full power. PEDOT Al4083 was spun-coated and annealed on a hotplate at 120 ˚C for 15 min to give a 30 nm film. Emitting layer was deposited from chloroform:chlorobenzene (95:5 v/v) solution (20 mg mL^{-1} total solids content). The dopant was dissolved in the solution of blend host in order to obtain final 5-12% concentration in the emitting layer. All solutions were filtrated directly before application using a PVDF (organic solvents) and PES (PEDOT Al4083) syringe filter with 0.45 µm pore size. All other layers were thermally evaporated using Kurt J. Lesker Spectros II deposition system at 10^{-6} mbar base pressure. All organic materials and aluminium were deposited at a rate of 1 Å s^{-1}. The LiF layer was deposited at a rate of 0.1–0.2 Å s^{-1}. Characterisation of OLED devices was conducted in a 10 inch integrating sphere (Labsphere) connected to a Source Measure Unit (SMU) Keithley 2400 and coupled with a spectrometer USB4000 (Ocean Optics). Further details are available in reference [19].

2. Synthesis

Proligand H_2L

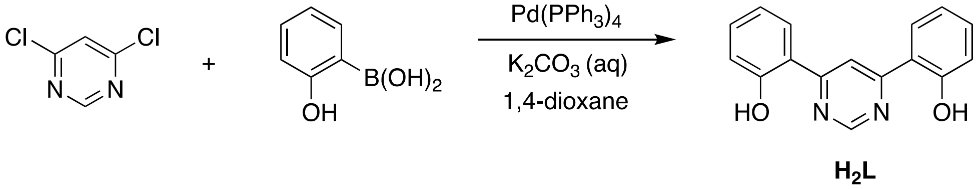

4,6-Dichloropyrimidine (2.35 g, 15.8 mmol) and 2-hydroxybenezenboronic acid (5.23 g, 37.9 mmol) were dissolved in 1,4-dioxane (90 mL). An aqueous solution of K_2CO_3 (2 M, 37 mL) was added and the contents of the flask were deoxygenated by passing a stream of argon through the vigorously stirred emulsion for 25 min. $Pd(PPh_3)_4$ (912 mg, 789 μmol) was introduced under argon flow and the reaction mixture was stirred at 95°C under argon atmosphere for 20 h. The reaction mixture was cooled to room temperature and water (200 mL) was added. The resultant precipitate was filtered off and washed with water followed by petroleum ether to give **H_2L** as a pale-yellow solid (2.80 g, 67%). ^1H NMR ($CDCl_3$ + DMSO-d_6, 400 MHz) δ_H = 9.02 (s, 1H), 8.26 (s, 1H), 7.90 (d, J = 7.8 Hz, 2H), 7.37 (app t, 2H), 7.01 (d, J = 8.3 Hz, 2H), 6.94 (app t, 2H), 2.29 (2 × OH +H_2O). ^{13}C NMR ($CDCl_3$ + DMSO-d_6, 101 MHz) δ_C = 164.8 (C), 161.0 (C), 153.7 (CH), 134.0 (CH), 127.0 (CH), 119.5 (CH), 119.2 (CH), 116.8 (C), 108.9 (CH).

Complex 1

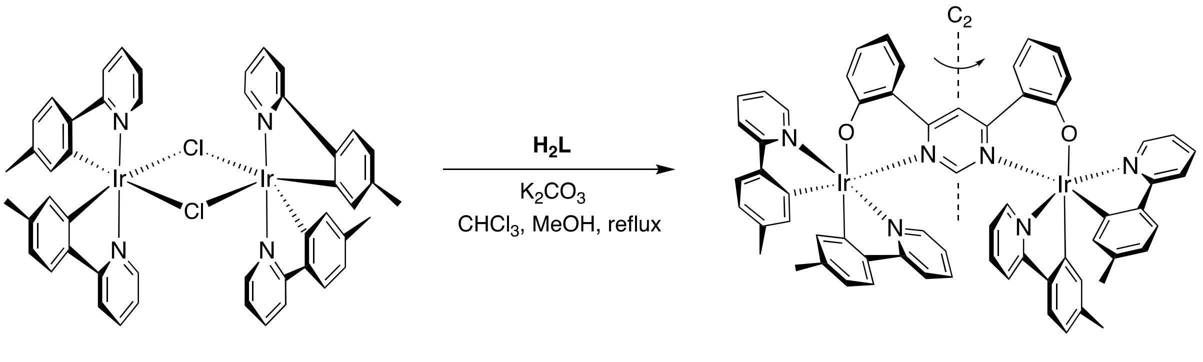

[Ir(Meppy)$_2$(μ-Cl)]$_2$

[Ir(Meppy)$_2$]$_2$(μ-**L**)

1

K_2CO_3 (98 mg, 704 μmol) was added to a solution of **H_2L** (47 mg, 176 μmol) and [**Ir(Meppy)$_2$(μ-Cl)]$_2$** (199 mg, 176 μmol) in a mixture of methanol (25 mL) and chloroform (5 mL), and the mixture was heated to reflux for 20 h under argon atmosphere. The solvents were then removed under reduced pressure. The resulting residue was suspended in DCM and the solid was filtered off. After washing with DCM, this material was purified by flash column chromatography on silica using DCM/acetone (gradient elution 100/0 → 80/20) to give complex **1** as a cherry-red solid (158 mg, 68%). ^1H NMR (CD_2Cl_2, 400 MHz) δ_H = 8.96 (d, J = 5.8 Hz, 2H), 7.96 (s, 1H), 7.84 (d, J = 5.7 Hz,

2H), 7.60 – 7.52 (m, 4H), 7.47 - 7.31 (m, 10H), 7.03 (app t, 2H), 6.99 (s, 1H), 6.85 – 6.79 (m, 4H), 6.62 (app d, 4H), 6.35 (app t, 2H), 6.09 (d, *J* = 8.2 Hz, 2H), 5.69 (s, 2H), 5.52 (s, 2H), 2.05 (s, 6H), 1.97 (s, 6H). ^{13}C NMR (CD_2Cl_2, 101 MHz) δ_C = 171.7 (C), 167.7 (C), 161.9 (C), 160.6 (CH), 150.7 (CH), 150.2 (C), 149.0 (C), 148.1 (C), 142.3 (C), 141.4 (C), 139.0 (C), 138.7 (C), 136.6 (CH), 136.2 (CH), 133.4 (CH), 132.8 (CH), 132.5 (CH), 129.8 (CH), 126.7 (CH), 125.9 (C), 123.6 (CH), 122.9 (CH), 122.7 (CH), 121.5 (CH), 121.0 (CH), 119.6 (CH), 119.1 (CH), 118.0 (CH), 115.4 (CH), 21.9 (CH_3), 21.7 (CH_3). Elemental (CHN) analysis: calc. for $C_{64}H_{50}Ir_2N_6O_2$, %: C, 58.25; H 3.82; N, 6.37. Found, %: C, 58.10; H, 3.81; N, 6.33.

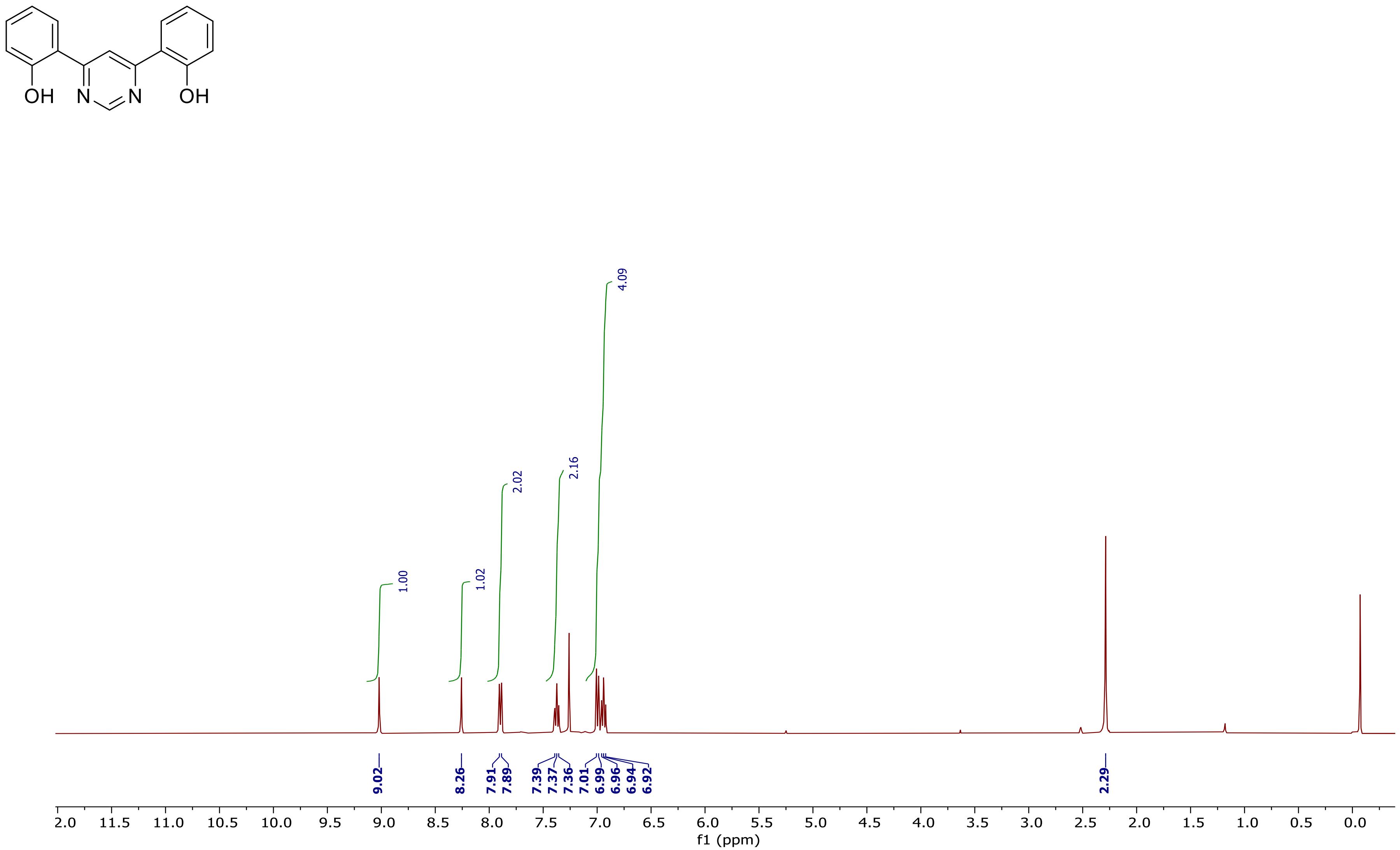

Figure S2.1. ^{1}H NMR spectrum of the proligand **H_2L** in $CDCl_3$.

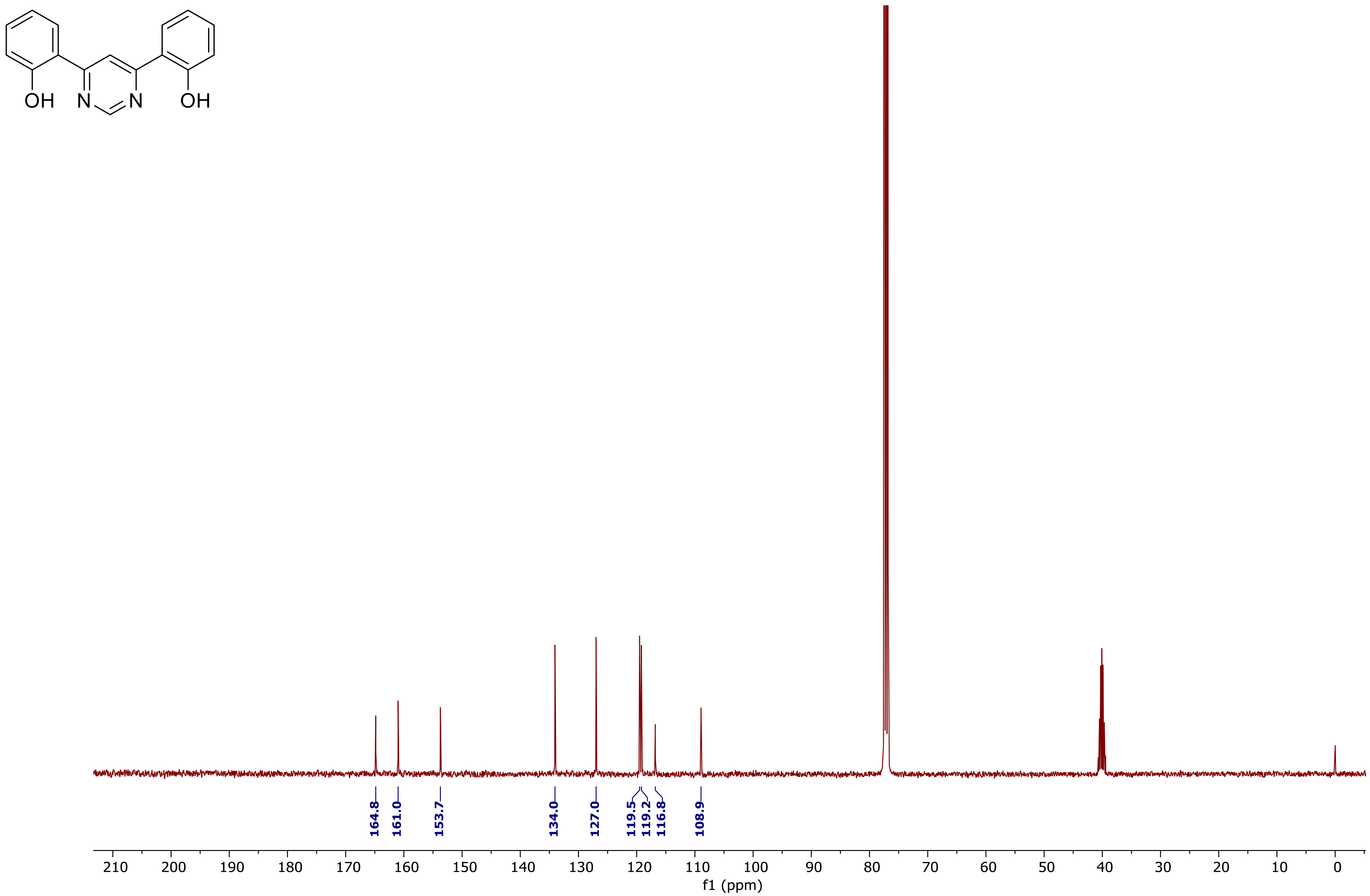

Figure S2.2. ^{13}C NMR spectrum of the proligand $\mathbf{H_2L}$ in $CDCl_3$.

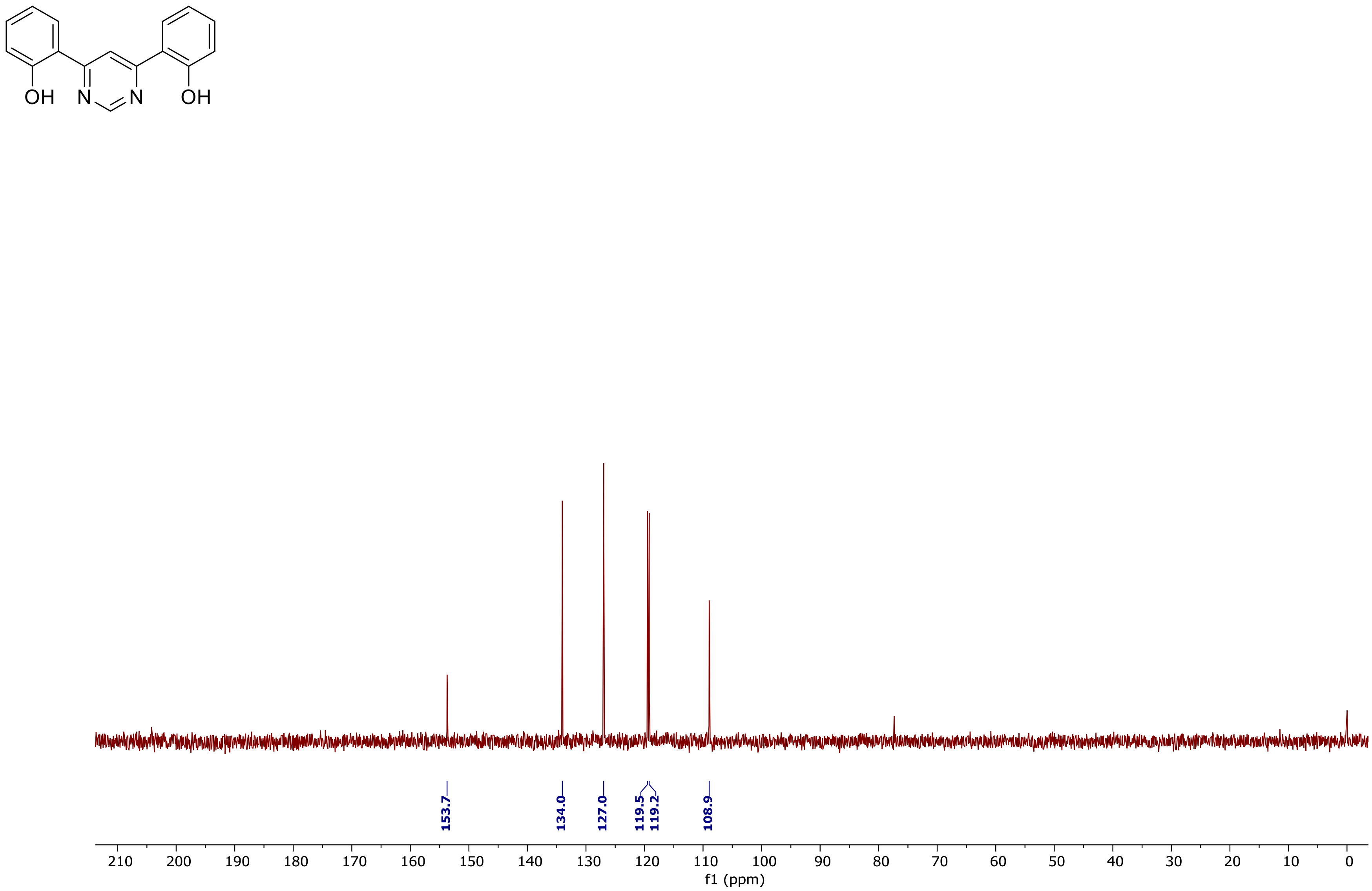

Figure S2.3. ^{13}C (DEPT135) NMR spectrum of the proligand $\mathbf{H_2L}$ in $CDCl_3$.

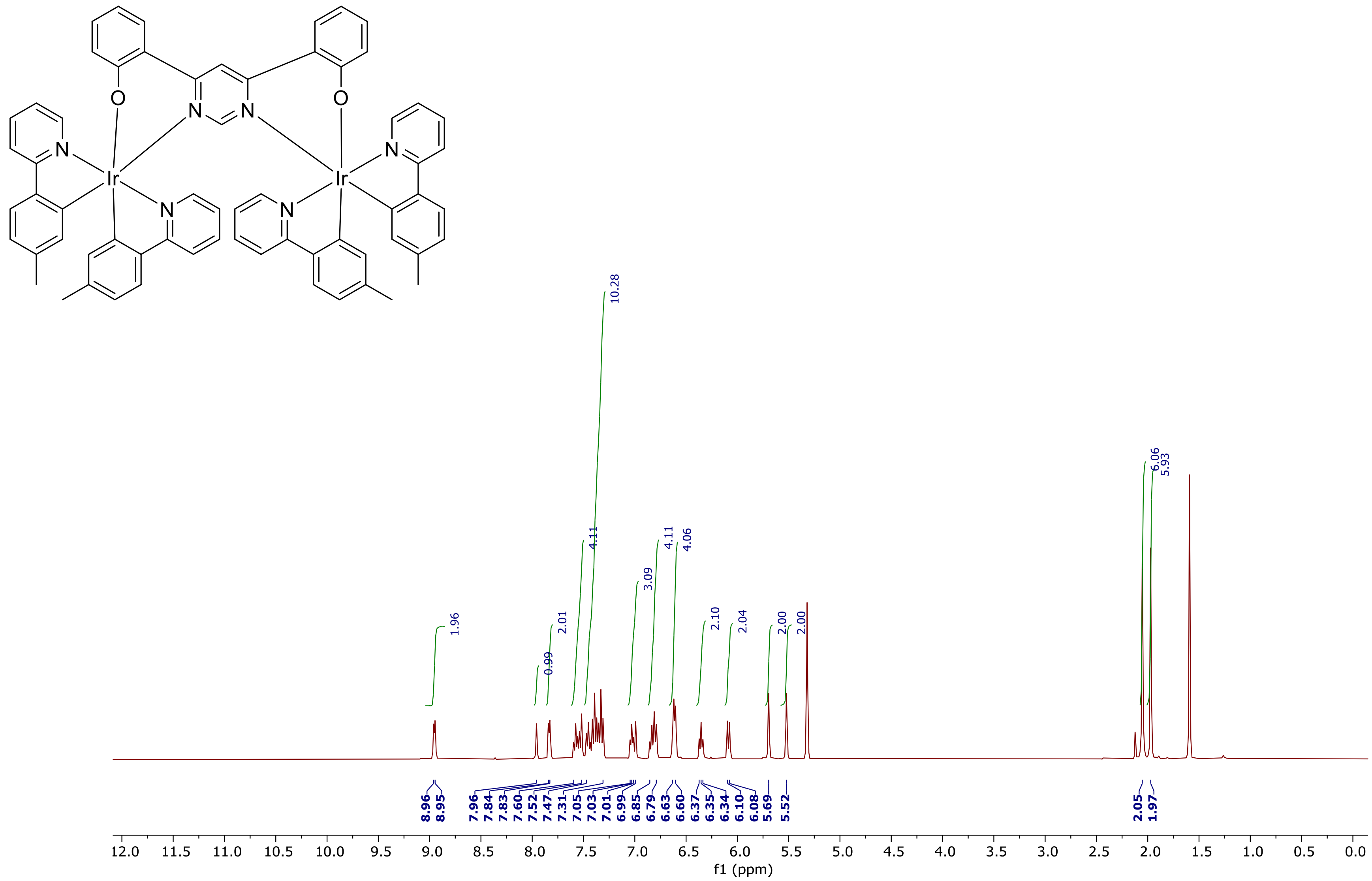

Figure S2.4. ^{1}H NMR spectrum of the complex **1** in $CDCl_3$.

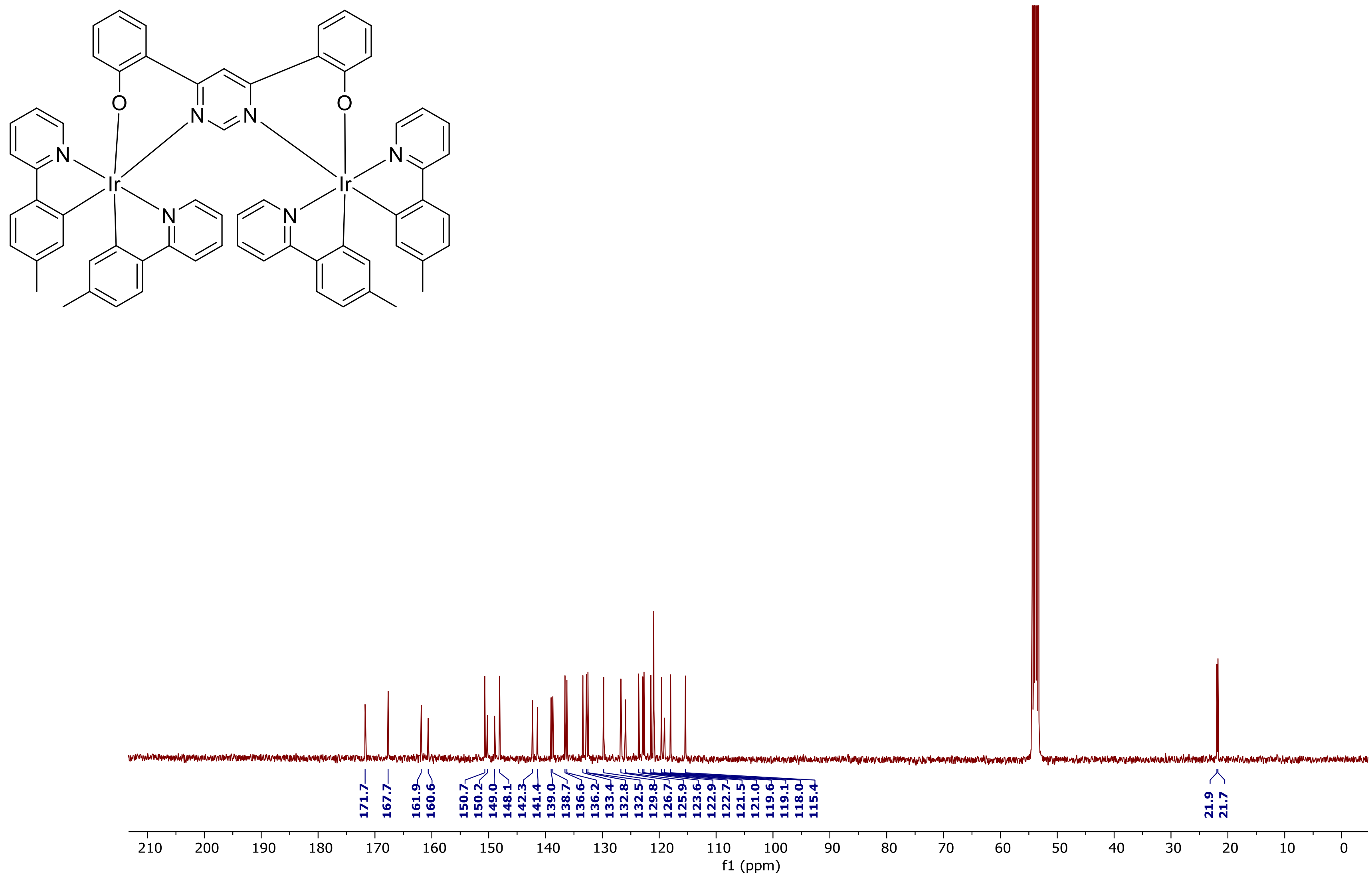

Figure S2.5. ^{13}C NMR spectrum of the complex **1** in $CDCl_3$.

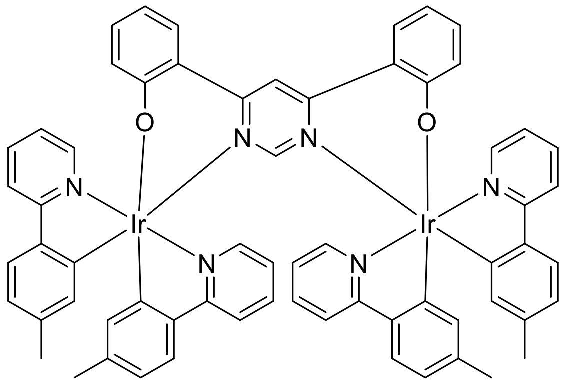

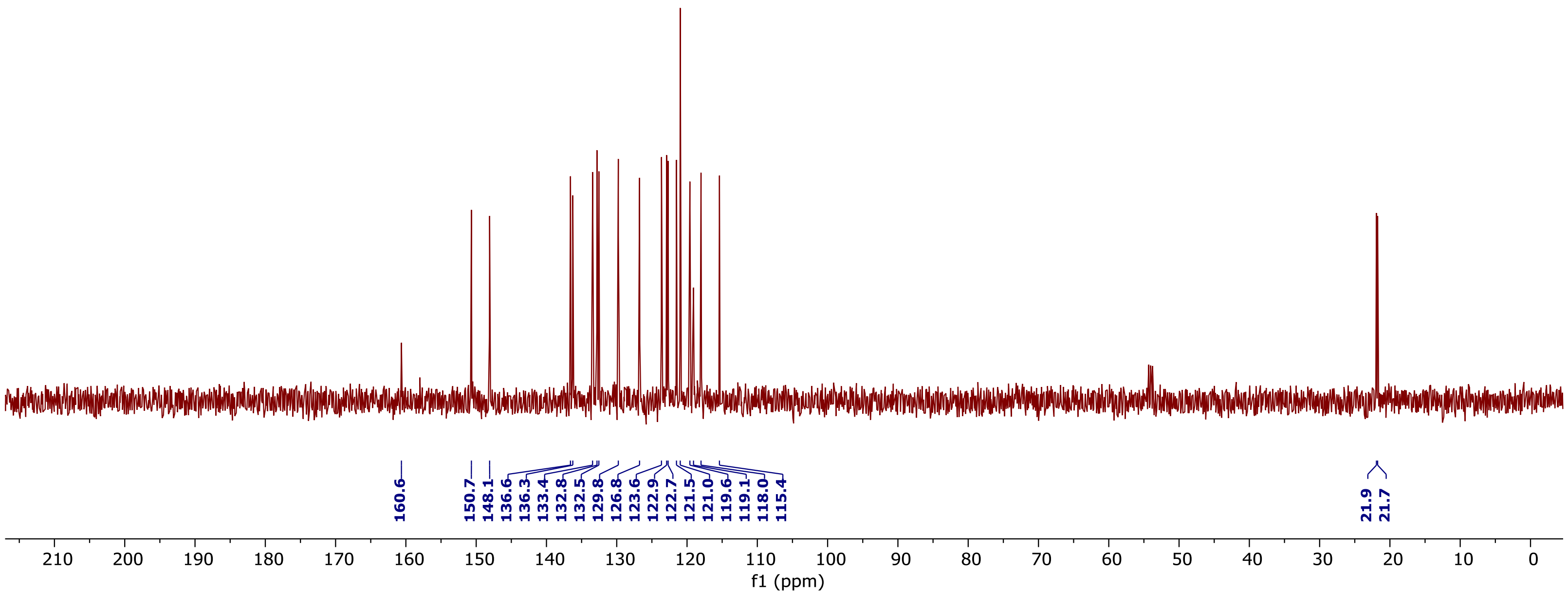

Figure S2.6. ^{13}C (DEPT 135) NMR spectrum of the complex **1** in $CDCl_3$.

3. X-ray crystallography

The X-ray single crystal data for complex **1** were collected at a temperature of 130.0 K using MoKα radiation (λ =0.71073Å) on a Bruker D8 Venture (Photon III MM C14 CPAD detector, IμS-3-microsource, focusing mirrors) 3-circle diffractometer equipped with a Cryostream (Oxford Cryosystems) open-flow nitrogen cryostat. The structure was solved by a direct method and refined by full-matrix least squares on F^2 for all data using Olex2[20] and SHELXTL[21] software. All non-hydrogen atoms were refined in anisotropic approximation: hydrogen atoms were placed in the calculated positions and refined in riding mode. Crystal data and parameters of refinement are listed in Table 3.1 below, and bond lengths and angles in Tables 3.2 and 3.3. The data have been deposited with the Cambridge Crystallographic Data Centre as supplementary publication CCDC 2288521.

Table 3.1 Crystal data and structure refinement parameters for complex **1**.

Empirical formula	$C_{64}H_{50}Ir_2N_6O_2$
Formula weight	1319.50
Temperature/K	130.0
Crystal system	monoclinic
Space group	C2/c
a/Å	30.2193(13)
b/Å	12.6557(5)
c/Å	14.4652(6)
α/°	90
β/°	115.1202(14)
γ/°	90
Volume/Å^3	5008.9(4)
Z	4
ρ_{calc}g/cm^3	1.750
μ/mm^{-1}	5.362
F(000)	2584.0
Crystal size/mm^3	0.045 × 0.032 × 0.006
Radiation	MoKα (λ = 0.71073)
2Θ range for data collection/°	4.28 to 55.996
Index ranges	$-39 \leq h \leq 39, -16 \leq k \leq 16, -19 \leq l \leq 19$
Reflections collected	52134
Independent reflections	6047 [R_{int} = 0.1281, R_{sigma} = 0.0757]
Data/restraints/parameters	6047/0/337
Goodness-of-fit on F^2	1.030
Final R indexes [I>=2σ (I)]	R_1 = 0.0416, wR_2 = 0.0694
Final R indexes [all data]	R_1 = 0.0765, wR_2 = 0.0790
Largest diff. peak/hole / e Å^{-3}	1.00/-1.48

Table 3.2 Bond lengths in complex **1**.

Atom	Atom	Length/Å
Ir1	O1	2.149(4)
Ir1	N1	2.183(4)
Ir1	N2	2.043(5)
Ir1	N3	2.023(5)
Ir1	C16	2.011(6)
Ir1	C28	1.997(6)
O1	C5	1.313(7)
N1	C1	1.341(6)
N1	C2	1.355(7)
N2	C10	1.343(7)
N2	C14	1.386(7)
N3	C22	1.343(8)
N3	C26	1.366(8)
C2	C3	1.385(7)
C2	C4	1.471(8)
C4	C5	1.410(8)
C4	C9	1.410(9)
C5	C6	1.409(8)
C6	C7	1.371(9)
C7	C8	1.400(9)
C8	C9	1.369(8)
C10	C11	1.368(8)
C11	C12	1.401(9)

Atom	Atom	Length/Å
C12	C13	1.377(9)
C13	C14	1.384(8)
C14	C15	1.453(8)
C15	C16	1.400(8)
C15	C20	1.402(8)
C16	C17	1.401(8)
C17	C18	1.385(8)
C18	C19	1.403(9)
C18	C21	1.496(9)
C19	C20	1.375(9)
C22	C23	1.379(9)
C23	C24	1.370(9)
C24	C25	1.368(9)
C25	C26	1.394(8)
C26	C27	1.453(8)
C27	C28	1.425(8)
C27	C32	1.394(9)
C28	C29	1.398(9)
C29	C30	1.405(8)
C30	C31	1.396(9)
C30	C33	1.496(9)
C31	C32	1.375(9)

Table 3.3 Bond angles in complex **1**.

Atom	Atom	Atom	Angle/°
O1	Ir1	N1	81.31(17)
N2	Ir1	O1	90.61(18)
N2	Ir1	N1	97.17(19)
N3	Ir1	O1	91.93(18)
N3	Ir1	N1	87.04(19)
N3	Ir1	N2	175.4(2)
C16	Ir1	O1	86.8(2)
C16	Ir1	N1	167.9(2)
C16	Ir1	N2	80.6(2)
C16	Ir1	N3	95.7(2)
C28	Ir1	O1	172.4(2)
C28	Ir1	N1	100.9(2)
C28	Ir1	N2	96.3(2)
C28	Ir1	N3	80.9(2)
C28	Ir1	C16	91.2(2)
C5	O1	Ir1	113.8(4)
C1	N1	Ir1	120.9(4)
C1	N1	C2	117.2(5)
C2	N1	Ir1	121.4(4)
C10	N2	Ir1	126.3(4)
C10	N2	C14	118.5(5)
C14	N2	Ir1	115.1(4)
C22	N3	Ir1	124.0(4)
C22	N3	C26	120.3(5)
C26	N3	Ir1	115.6(4)
N1	C1	N1[1]	126.7(8)
N1	C2	C3	118.3(6)
N1	C2	C4	122.3(5)
C3	C2	C4	119.3(6)
C2[1]	C3	C2	122.2(8)
C5	C4	C2	120.8(5)
C9	C4	C2	119.3(5)
C9	C4	C5	119.8(5)
O1	C5	C4	122.8(5)
O1	C5	C6	119.2(5)
C6	C5	C4	117.9(5)
C7	C6	C5	121.3(6)
C6	C7	C8	120.5(6)
C9	C8	C7	119.6(6)
C8	C9	C4	120.9(6)
N2	C10	C11	123.0(6)
C10	C11	C12	119.2(6)
C13	C12	C11	118.4(6)
C12	C13	C14	120.7(6)
N2	C14	C15	112.8(5)
C13	C14	N2	120.1(6)
C13	C14	C15	127.0(6)
C16	C15	C14	116.8(5)
C16	C15	C20	119.7(6)
C20	C15	C14	123.4(5)
C15	C16	Ir1	113.5(4)
C15	C16	C17	118.4(5)
C17	C16	Ir1	127.6(5)
C18	C17	C16	122.1(6)
C17	C18	C19	118.4(6)
C17	C18	C21	121.5(6)
C19	C18	C21	120.0(6)
C20	C19	C18	120.7(6)
C19	C20	C15	120.6(6)
N3	C22	C23	121.3(6)
C24	C23	C22	119.2(6)
C25	C24	C23	119.6(7)
C24	C25	C26	120.4(6)
N3	C26	C25	119.0(6)
N3	C26	C27	114.4(5)
C25	C26	C27	126.5(6)
C28	C27	C26	114.5(6)
C32	C27	C26	124.6(6)
C32	C27	C28	120.9(6)
C27	C28	Ir1	113.9(4)
C29	C28	Ir1	129.4(5)
C29	C28	C27	116.7(6)
C28	C29	C30	123.1(6)
C29	C30	C33	121.0(6)
C31	C30	C29	117.5(6)
C31	C30	C33	121.5(6)
C32	C31	C30	121.8(6)
C31	C32	C27	119.9(6)

[1]1-X,+Y,3/2-Z

4. Theory

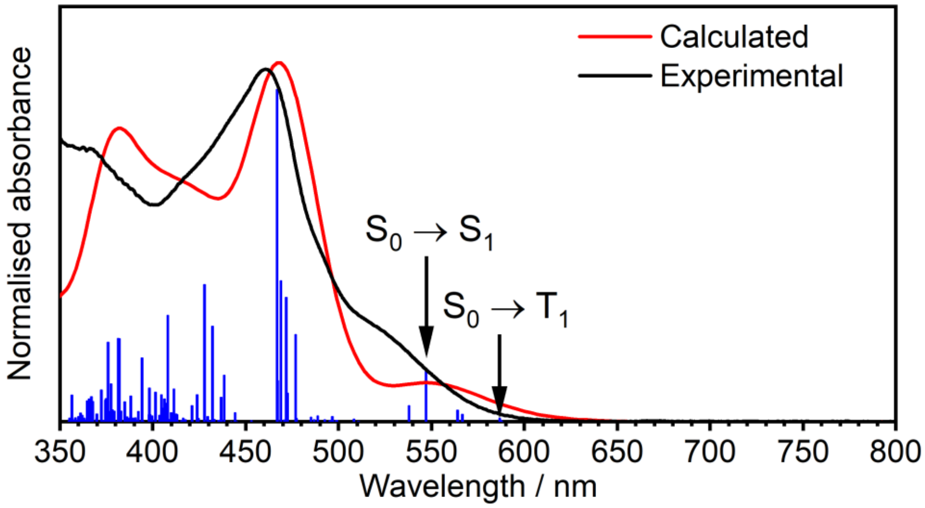

Figure S4.1. Simulated absorption spectrum of **1** calculated at the S_0 geometry.

Table S4.1. Summary of the eight lowest spin-orbit coupling (SOC) excited states in complex **1** at the T_1 geometry.

SOC state	Dominant composition[a]	Assignment	Energy, eV	Oscillator strength	Radiative rate constant k_r, s^{-1}	Boltzmann factor (T = 295 K)[b]
**1**	88.9% T_1	T_1	1.7471	0.000188	5.6×10^4	-
**2**	88.7% T_1, 2.0% S_1		1.7481	0.000252	7.5×10^4	0.9608
**3**	89.2% T_1		1.7510	0.002906	8.7×10^5	0.8672
**4**	80.6% S_1, 4.1% T_2	S_1	1.8162	0.015177	4.9×10^6	0.0659
**5**	86.8% T_2, 3.0% T_3	T_2	1.9061	0.000283	1.0×10^5	0.0019
**6**	85.4% T_2, 3.6% S_1		1.9072	0.001139	4.0×10^5	0.0018
**7**	87.8% T_2		1.9119	0.004208	1.5×10^6	0.0015
**8**	81.9% S_2, 5.1% T_3	S_2	2.0102	0.014166	5.6×10^6	< 0.0001

[a] Listing only states with ≥ 2% contribution; [b] Product of the expression $\exp(\Delta E_{1,n} / RT)$ for T = 295 K, where $\Delta E_{1,n}$ is the energy difference between the SOC states 1 and n.

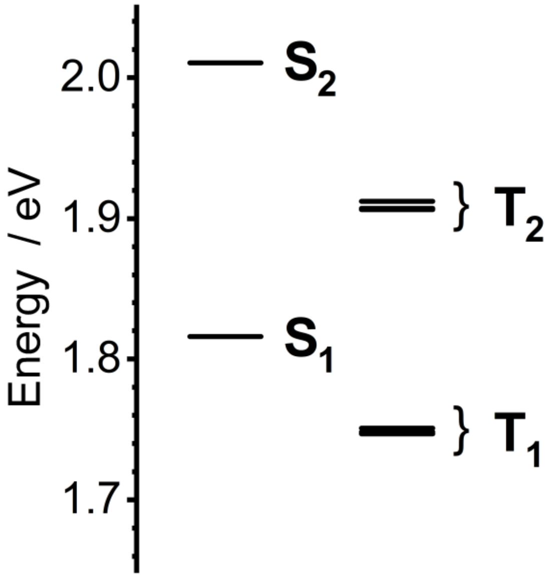

Figure S4.2. Energy of the eight lowest spin-orbit coupling (SOC) excited states in complex **1** at the T_1 geometry presented in a graphical form.

5. Photophysics

a) Solution state

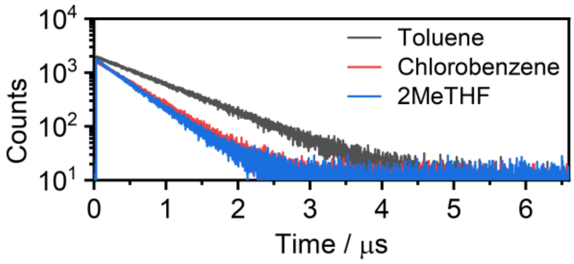

Figure S5.1. Photoluminescence decay traces recorded in degassed dilute (10^{-5} M) solutions of **1** in solvents indicated in figure legend, at room temperature.

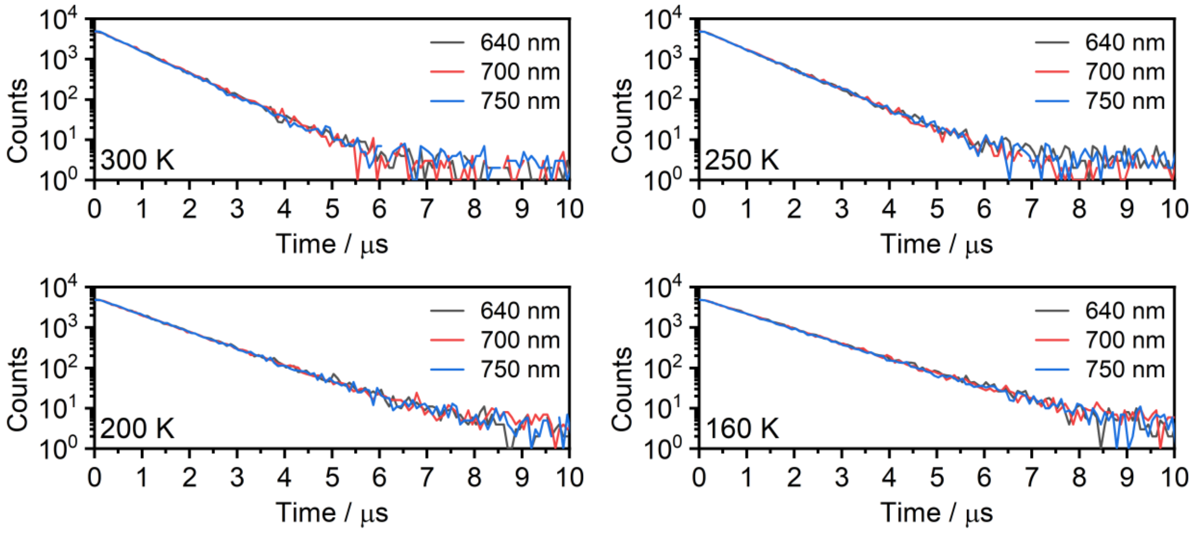

Figure S5.2. Photoluminescence decay traces recorded in degassed dilute (10^{-5} M) solution of **1** in toluene, at temperatures indicated in each figure legend.

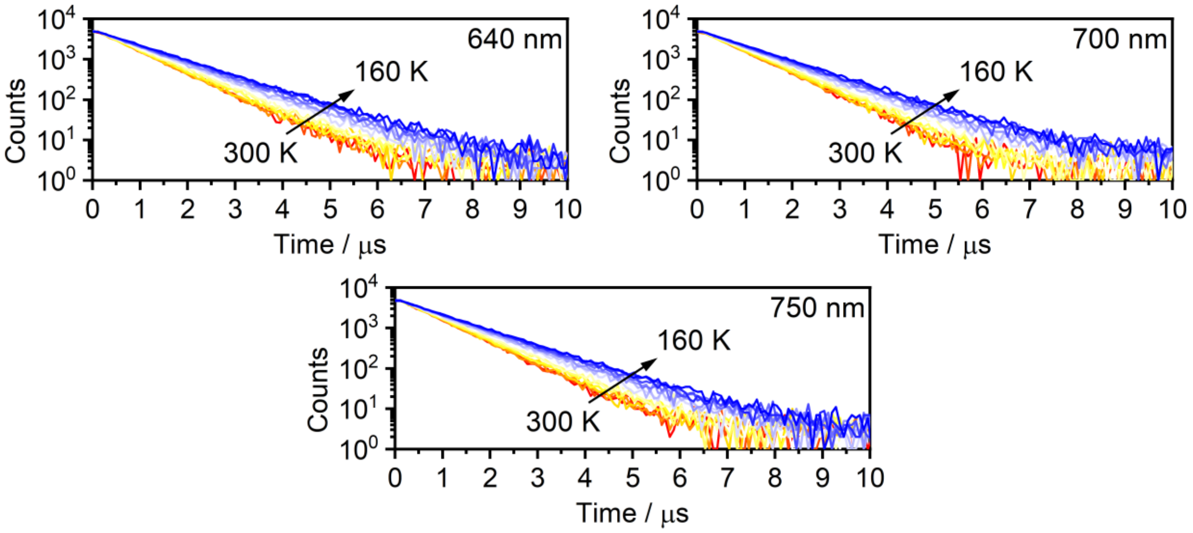

Figure S5.3. Photoluminescence decay traces recorded in degassed dilute (10^{-5} M) solution of **1** in toluene, at temperatures from 160 to 300 K at collection wavelengths indicated in each figure legend.

b) Solid film (polymer matrix)

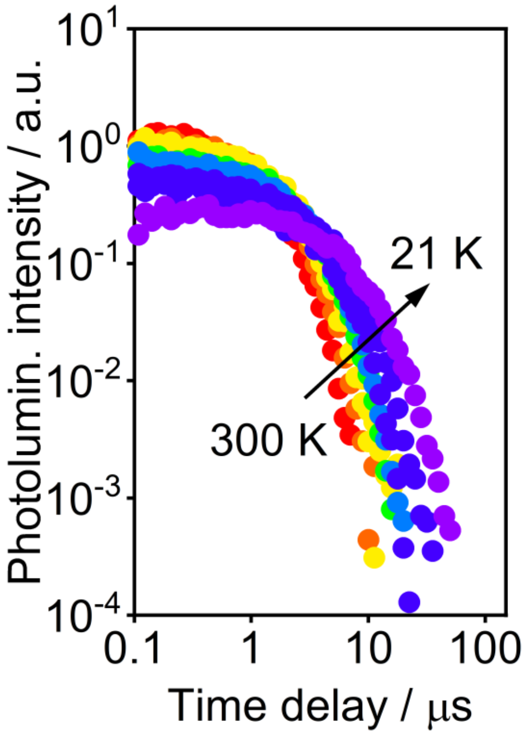

Figure S5.4. Photoluminescence decay traces recorded in dilute polystyrene film of **1** (0.1 % w/w) in vacuum at temperatures from 21 to 300 K.

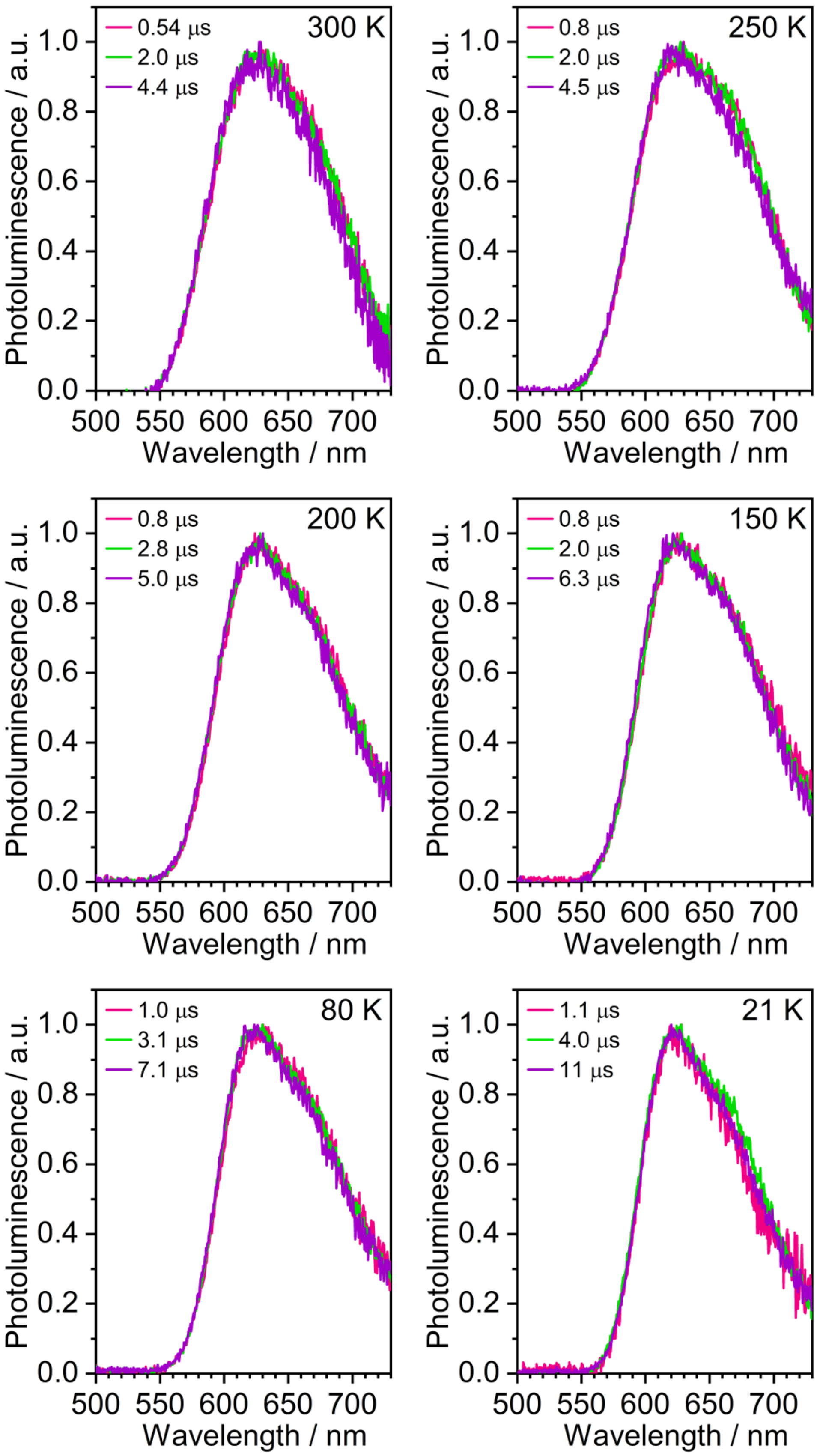

Figure S5.5. Time-resolved photoluminescence spectra recorded in dilute polystyrene film of **1** (0.1 % w/w) in vacuum at temperatures and delay times indicated in each figure legend.

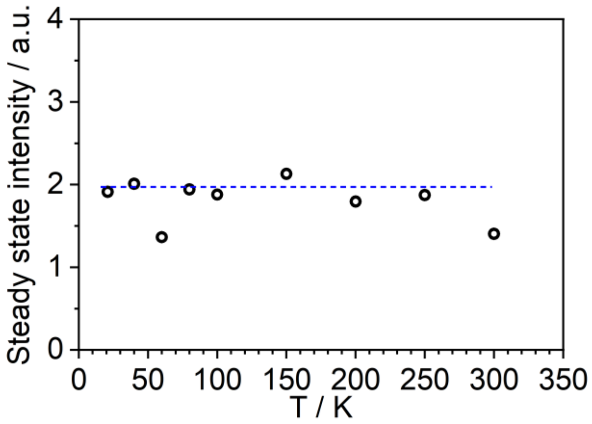

Figure S5.6. Steady-state photoluminescence intensity recorded in dilute polystyrene film of **1** (0.1 % w/w) as a function of temperature.

6. Electrochemistry

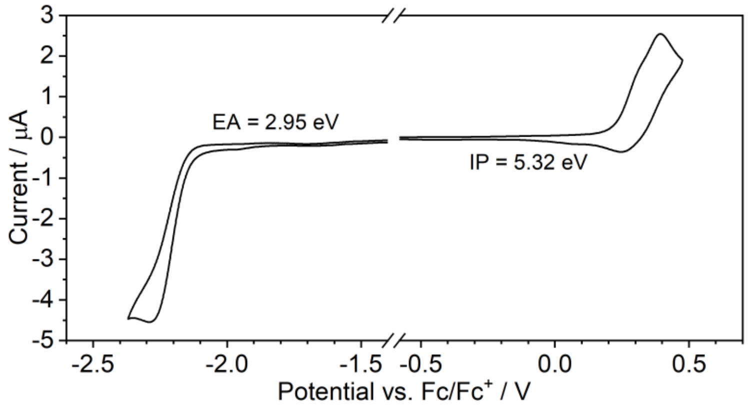

Figure S6.1 Cyclic voltammogram (CV) of **1** (c = 10^{-3} M) in 0.1 M Bu_4NBF_4 / CH_2Cl_2 solution.

Complex **1** displays partly reversible oxidation and irreversible reduction processes. Interestingly, the anodic half wave of the oxidation process is composed of two signals, indicating only a small potential difference between formation of cations and di-cations in the process. This behaviour of the oxidation half wave may suggest sequential oxidation of the two coordinated metal centres, rather than them oxidising together at once.

7. OLED devices

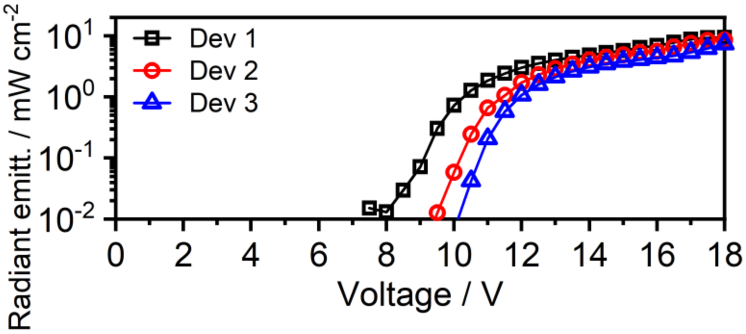

Figure S7.1. Radiant emittance-voltage (J-V) characteristics of devices 1-3.

8. References

1 M. Roemelt, D. Maganas, S. DeBeer and F. Neese, *J. Chem. Phys.*, 2013, **138**, 204101.

2 B. de Souza, G. Farias, F. Neese and R. Izsák, *J. Chem. Theory Comput.*, 2019, **15**, 1896–1904.

3 E. van Lenthe, E. J. Baerends and J. G. Snijders, *J. Chem. Phys.*, 1993, **99**, 4597–4610.

4 E. van Lenthe, E. J. Baerends and J. G. Snijders, *J. Chem. Phys.*, 1994, **101**, 9783–9792.

5 F. Neese, *WIREs Comput. Mol. Sci.*, 2012, **2**, 73–78.

6 F. Neese, *WIREs Comput. Mol. Sci.*, , DOI:10.1002/wcms.1606.

7 A. D. Becke, *Phys. Rev. A*, 1988, **38**, 3098–3100.

8 F. Weigend and R. Ahlrichs, *Phys. Chem. Chem. Phys.*, 2005, **7**, 3297.

9 A.-R. Allouche, *J. Comput. Chem.*, 2011, **32**, 174–182.

10 F. Weigend, *Phys. Chem. Chem. Phys.*, 2006, **8**, 1057.

11 S. Grimme, S. Ehrlich and L. Goerigk, *J. Comput. Chem.*, 2011, **32**, 1456–1465.

12 S. Grimme, J. Antony, S. Ehrlich and H. Krieg, *J. Chem. Phys.*, 2010, **132**, 154104.

13 D. A. Pantazis, X. Y. Chen, C. R. Landis and F. Neese, *J. Chem. Theory Comput.*, 2008, **4**, 908–919.

14 P. Data, P. Pander, M. Lapkowski, A. Swist, J. Soloducho, R. R. Reghu and J. V. Grazulevicius, *Electrochim. Acta*, 2014, **128**, 430–438.

15 P. Pander, P. Data, R. Turczyn, M. Lapkowski, A. Swist, J. Soloducho and A. P. Monkman, *Electrochim. Acta*, 2016, **210**, 773–782.

16 C. M. Cardona, W. Li, A. E. Kaifer, D. Stockdale and G. C. Bazan, *Adv. Mater.*, 2011, **23**, 2367–2371.

17 J.-L. Bredas, *Mater. Horiz.*, 2014, **1**, 17–19.

18 P. Pander, P. Data and F. B. Dias, *J. Vis. Exp.*, , DOI:10.3791/56614.

19 D. de Sa Pereira, A. P. Monkman and P. Data, *J. Vis. Exp.*, , DOI:10.3791/56593.

20 O. V. Dolomanov, L. J. Bourhis, R. J. Gildea, J. A. K. Howard and H. Puschmann, *J. Appl. Crystallogr.*, 2009, **42**, 339–341.

21 G. M. Sheldrick, *Acta Crystallogr. Sect. A Found. Crystallogr.*, 2008, **64**, 112–122.
